# Gut microbiome variation modulates the effects of dietary fiber on host metabolism

**DOI:** 10.1186/s40168-021-01061-6

**Published:** 2021-05-20

**Authors:** Sofia M. Murga-Garrido, Qilin Hong, Tzu-Wen L. Cross, Evan R. Hutchison, Jessica Han, Sydney P. Thomas, Eugenio I. Vivas, John Denu, Danilo G. Ceschin, Zheng-Zheng Tang, Federico E. Rey

**Affiliations:** 1 Department of Bacteriology, University of Wisconsin-Madison, 1550 Linden Dr., Madison, WI 53706 USA; 2 PECEM (MD/PhD), Facultad de Medicina, Universidad Nacional Autónoma de México, Coyoacán, Ciudad de México, México; 3 Department of Biostatistics and Medical Informatics, University of Wisconsin-Madison, 600 Highland Avenue, Madison, WI 53792 USA; 4 Present Address: Department of Nutrition Science, Purdue University, 700 W. State Street, Stone Hall 205, West Lafayette, IN 47907 USA; 5 Wisconsin Institute for Discovery, Madison, WI USA; 6 Unidad de Bioinformática Traslacional, Centro de Investigación en Medicina Traslacional Severo Amuchástegui, Instituto Universitario de Ciencias Biomédicas de Córdoba, Av. Naciones Unidas 420, 5000 Córdoba, CP Argentina

## Abstract

**Background:**

There is general consensus that consumption of dietary fermentable fiber improves cardiometabolic health, in part by promoting mutualistic microbes and by increasing production of beneficial metabolites in the distal gut. However, human studies have reported variations in the observed benefits among individuals consuming the same fiber. Several factors likely contribute to this variation, including host genetic and gut microbial differences. We hypothesized that gut microbial metabolism of dietary fiber represents an important and differential factor that modulates how dietary fiber impacts the host.

**Results:**

We examined genetically identical gnotobiotic mice harboring two distinct complex gut microbial communities and exposed to four isocaloric diets, each containing different fibers: (i) cellulose, (ii) inulin, (iii) pectin, (iv) a mix of 5 fermentable fibers (assorted fiber). Gut microbiome analysis showed that each transplanted community preserved a core of common taxa across diets that differentiated it from the other community, but there were variations in richness and bacterial taxa abundance within each community among the different diet treatments. Host epigenetic, transcriptional, and metabolomic analyses revealed diet-directed differences between animals colonized with the two communities, including variation in amino acids and lipid pathways that were associated with divergent health outcomes.

**Conclusion:**

This study demonstrates that interindividual variation in the gut microbiome is causally linked to differential effects of dietary fiber on host metabolic phenotypes and suggests that a one-fits-all fiber supplementation approach to promote health is unlikely to elicit consistent effects across individuals. Overall, the presented results underscore the importance of microbe-diet interactions on host metabolism and suggest that gut microbes modulate dietary fiber efficacy.

Video abstract

**Supplementary Information:**

The online version contains supplementary material available at 10.1186/s40168-021-01061-6.

## Introduction

Humans harbor diverse and dynamic microbial communities in their intestines that span the three domains of life [1, 2]. These microbes play key roles on host biology, including breakdown of complex dietary components, vitamin production, energy harvesting, immune system maturation, and protection against pathogens [2–4]. While many microbial functions are shared among gut communities from unrelated individuals, large interpersonal differences have also been reported [1]. Factors such as genetics, environment, and lifestyles contribute to these differences [5, 6]. Identifying the consequences of this variation as it relates to host immune responses, drug effectiveness, and metabolism is key to better understand how microbes modulate human biology and for successful implementation of precision medicine and personalized nutritional strategies.

Metabolic disease represents a major health challenge worldwide, with an estimate prevalence of 20–25% of the world’s adult population [7, 8]. A large number of studies indicate that the gut microbiota influences the development of metabolic syndrome [9–13]. Gut microbes exacerbate metabolic disease in part by activating inflammatory pathways, and by producing compounds from diet that dysregulate host signaling and metabolism [14–17]. Microbes can also play protective roles against metabolic disease. A large body of evidence suggests that microbes and microbial metabolites derived from dietary fiber, including short-chain fatty acids (SCFAs), mediate some of the beneficial effects associated with dietary fiber consumption [18–20].

Dietary fiber are edible carbohydrate polymers with at least three monomeric units that are resistant to host digestive enzymes and not broken down or absorbed in the small intestine [21]. The chemical structure of a fiber determines important physicochemical properties including its solubility and viscosity. Dietary fibers can be divided into soluble and insoluble forms [22, 23]. Insoluble forms such as cellulose have a fecal bulking effect, and resist metabolization by gut microbes, particularly in monogastric hosts. Dietary fibers that microbes can use for carbon and energy are also referred as microbiota-accessible carbohydrates (MACs) [24]. MACs, such as inulin, pectin, and resistant starches, are broken down and metabolized through complex mechanisms by different gut bacteria [25]. Multiple lines of evidence suggest that dietary MACs have important effects on the ecology of the gut ecosystem [26]. MACs can support the growth of beneficial bacteria, promote intestinal barrier function, lower systemic inflammation [27], and prevent some of the detrimental effects caused by high-fat diet [28]. Microbial metabolism of MACs also promotes hepatic fatty acid metabolism at least in part via production of acetate, which serves as precursor for hepatic synthesis of fatty acids and related glycerophospholipid species [29].

While epidemiological studies support the notion that consumption of dietary fiber is generally beneficial for metabolic and cardiovascular health [30], results of interventions in humans vary widely. Several studies indicate that there is a large degree of interpersonal variation in the benefits attained among individuals receiving the same dietary fiber intervention [31–36]. In some cases, these inconsistent effects of fiber on host metabolism across subjects have been linked to differences in the gut microbiota of the consumers [32, 36] and potential microbial biomarkers for responsiveness to specific dietary interventions have been identified [34, 35, 37]. One study identified a higher *Prevotella*/*Bacteroides* ratio associated with improved glucose homeostasis in response to barley kernels in humans and demonstrated that *Prevotella copri* modulates glucose homeostasis in mice [35].

The studies described above suggest that interindividual differences in the gut microbiota may influence host metabolic responses to dietary fiber in humans. We sought to further examine this hypothesis using a tractable animal model and defined dietary fiber interventions. We colonized genetically identical germ-free (GF) mice with two distinct human fecal communities and fed them isocaloric diets containing different types of fiber. We found that the two transplanted communities elicited divergent metabolic epigenetic and transcriptional responses to the same dietary fiber. Furthermore, differences between mice colonized with these two communities varied depending on the type of fiber the animals consumed. Lastly, we identified candidate taxa and metabolites associated with these host phenotypes.

## Results and discussion

### Identifying fecal microbiomes with distinct metabolic potential

We sought to identify two human gut communities that upon engraftment in mice exhibit significantly distinct metabolic capacities. We used fecal specimens from a cohort of previously analyzed samples obtained from adults in their mid-seventies [38]. We initially selected eight fecal samples that showed significant compositional differences (Fig. S1A, B) and used them to colonize eight groups of adult male GF C57BL/6 mice (*n* = 2–4/fecal sample). Mice were fed a semi-purified diet containing an assortment of diverse, commonly consumed, commercially available fibers (i.e., assorted fiber) that included resistant starch (RS) type 2 and 4, short-chain fructo-oligosaccharides (scFOS), inulin, and pectin (total fiber content 10% w/w; Table S1). This diet—as well as all the other diets used in this study, were formulated to mimic human consumption while maintaining a defined and reproducible composition. We used 10% w/w dietary fiber and 35% kcal derived from fat as it is comparable to the intake level of dietary fiber in US adults from 2001 to 2010 based on the National Health and Nutrition Examination Survey (NHANES) data [39, 40]. This was calculated by gram of fiber per 1000 kcal intake estimated for humans and adjusted to typical mouse caloric consumption. Animals were placed on this diet 1 week prior to colonization and maintained for two additional weeks after inoculation. Comparison of 16S rRNA gene analysis of cecal samples obtained from transplanted animals clustered by donor (Fig. S1C). Transplanted bacterial communities were more similar to that of their donor than to any other human sample in the dataset (Fig. S1D). Recovery of genera of at least 0.02% relative abundance in at least one of the samples associated with each human subject (i.e., donor and mouse fecal pellets) was 59.2% ± 10.8 (Fig. S1E) and accounted for 85 ± 14% of the relative abundance of the community from the donor (Fig. S1F). The transplanted communities also exhibited differences in alpha diversity (Fig. S1G). Furthermore, as expected from this variation, transplanted communities also differed in their capacity to produce SCFAs (Fig. S2). There was a ~ 4-fold range in the levels of cecal butyrate among the eight groups despite all animals consuming the same diet (Fig. S2A). Butyrate is known to vary widely among humans and has been linked with beneficial health effects on the host [41, 42]. Additionally, we used PICRUSt2 to predict the functional profiles of the 8 transplanted communities using 16S rRNA gene data [43, 44]. Principal coordinates analysis (PCoA) using Bray Curtis dissimilarity (Fig. S3) shows clear separation among most communities suggesting distinct functional capabilities of engrafted microbiomes. Following these analyses, we selected two markedly different communities: samples 1 and 8, from here on referred as SubA (i.e., fecal community from subject A) and SubB (i.e., fecal community from subject B) respectively, based on differences in alpha diversity, predicted metabolic properties, and capacities to produce butyrate, to examine how variation in gut community composition modulate host responses to different types of fiber. The donors of these samples are overweight (BMI = 30), have no history of type II diabetes, cancer, or heart disease, and have self-reported consumption of a standard western-type diet.

### Effects of dietary fiber on host metabolic outcomes is influenced by gut microbial community

**Fig. 1:**
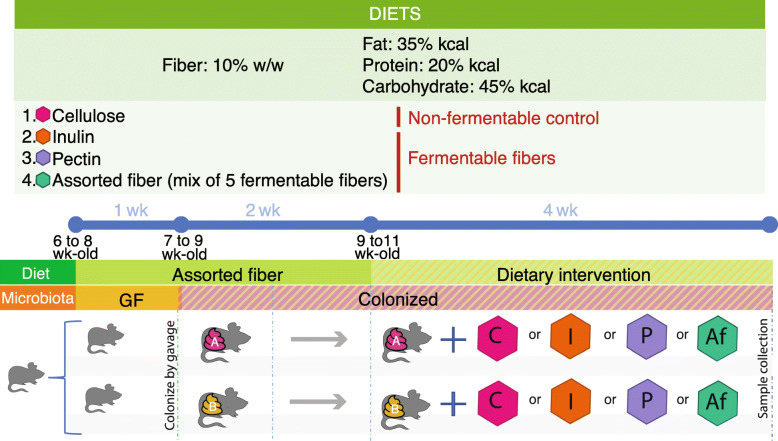
Study design. Six to eight-week-old C57BL/6 GF male mice were placed on irradiated diet containing a mix of five fibers (assorted fiber diet; Af); a week later mice were colonized with one of two different human fecal samples SubA, SubB. Bedding and wires with food were exchanged between cages of mice colonized with the same community to minimize cage effects. Two weeks after colonization gnotobiotic mice received one of four isocaloric diets that vary by the type of fiber (10% w/w): cellulose (C), inulin (I), pectin (P), and assorted fiber (Af). Mice were maintained in these diets for 4 weeks

Six- to eight-week-old male GF C57BL/6 mice were placed on the assorted fiber diet described above for 1 week, and subsequently colonized via oral gavage with fecal communities SubA or SubB (*n* = 30–36 mice/community, *n* = 66). Mice colonized with these two communities were maintained on the same diet for 2 weeks to allow the engrafted microbiomes to stabilize. After this stabilization period, mice colonized with each community were divided into four treatments (Fig. 1) and received one of four isocaloric diets (*n* = 7–10 mice/community/diet) that differed on the type of fiber they contained: (i) cellulose (non-fermentable fiber), (ii) inulin, (iii) pectin, or (iv) assorted fiber described above (Fig. 1). We selected cellulose as a source fiber that is mostly not accessible to microbes in the mouse gut. This diet served as a control, to define baseline differences between mice colonized with the two communities that were independent of interactions with MACs. We chose inulin and pectin as the former is commonly used as a prebiotic in the USA, while the latter has been proven to support growth of a wide variety of gut microbes [45], and it is commonly used as a dietary supplement. We also chose these two dietary fibers due to their distinct structures, including differences in basic units, linkages, and degree of polymerization. We colonized all mice in the same diet (assorted fiber diet) to favor consistent engraftment of mice inoculated with the same community. Furthermore, the assorted fiber diet has the same total amount of dietary fiber as the rest of the treatment groups used in this study, but with more diversity in fermentable substrates, which we reasoned would support engraftment of taxa relevant to all dietary treatments. Inclusion of this group in the experimental phase of the study also served as a control to inform whether this diet used during the colonization period drives major differences between mice colonized with the two communities used in the study.

**Fig. 2:**
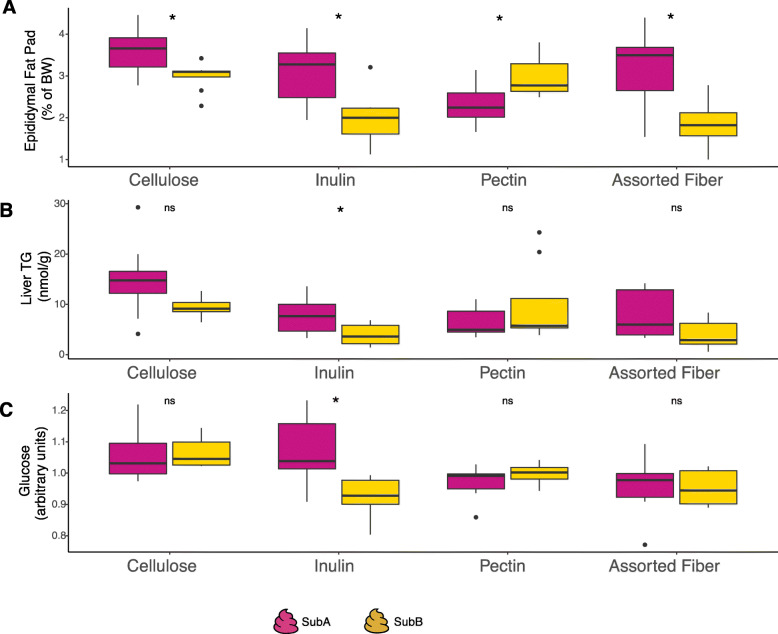
Gut microbiome impact on host metabolic phenotypes in different dietary fibers. Phenotypes were measured after 6 weeks colonization and 4 weeks of specific dietary fiber exposure (~ 15 weeks old). **a** Epididymal fat pad weight expressed a percentage of body weight (*n* = 7–10/community/diet). **b** Liver triglycerides levels (*n* = 7–10/community/diet). **c** Serum glucose levels (arbitrary units) as measured by UPLC/MS/MS (untargeted metabolomics platform; *n* = 6/community/diet). Wilcoxon rank sum test was conducted to examine whether two samples are likely to derive from the same population. Box plots represent median, interquartile range, minimum and maximum value in the data, and potential outliers. *P < 0.05, ns = not significant. SubA, magenta; SubB, yellow

We compared metabolic phenotypes of animals colonized with the two different communities consuming each of the diets described above (Table S2). We found significant effects of diet, gut community, and their interaction on host adiposity as determined by epididymal fat pad weight (normalized by body weight) (two-way ANOVA *P* < 0.01). Diet and community, but not their interaction, also showed significant effect on liver triglycerides (TG) (two-way ANOVA *P* < 0.05), whereas diet and its interaction with gut community showed a significant effect on serum glucose levels (two-way ANOVA *P* < 0.05; Table S2). Remarkably, while the pectin diet had an overall beneficial effect on metabolic phenotypes relative to non-fermentable cellulose (i.e., reduced adiposity and liver TG) for SubA-colonized mice, this diet was less favorable for SubB-colonized animals, which showed the strongest benefits on the inulin fiber (Fig. 2, Table S2). We also assessed whether there were significant differences in these phenotypes between mice colonized with the two different communities that were exposed to the same diet through pairwise comparisons using Wilcoxon rank sum test. In the cellulose diet, SubB-colonized mice showed lower levels of adiposity compared to mice colonized with SubA, whereas there were no statistical differences in the levels of liver TG and fasting serum glucose between these groups (Fig. 2). In the inulin diet, mice inoculated with SubB showed decreased adiposity, decreased liver TG, and lower serum levels of fasting glucose relative to animals colonized with SubA. In contrast, pectin-fed mice colonized with SubB accumulated more fat mass relative to SubA-colonized counterparts (Fig. 2a), whereas serum glucose and liver TG were comparable between the two community groups. Lastly, mice colonized with SubB showed significantly lower levels of adiposity than those colonized with SubA in the assorted fiber diet, whereas serum glucose and liver TG were comparable between the two groups (Fig. 2). Altogether, these results underscore the importance of microbe by dietary fiber interactions on host metabolism and suggest that gut microbes modulate responses to dietary fiber.

### Dietary fibers cause significant restructuring of SubA and SubB communities

**Fig. 3:**
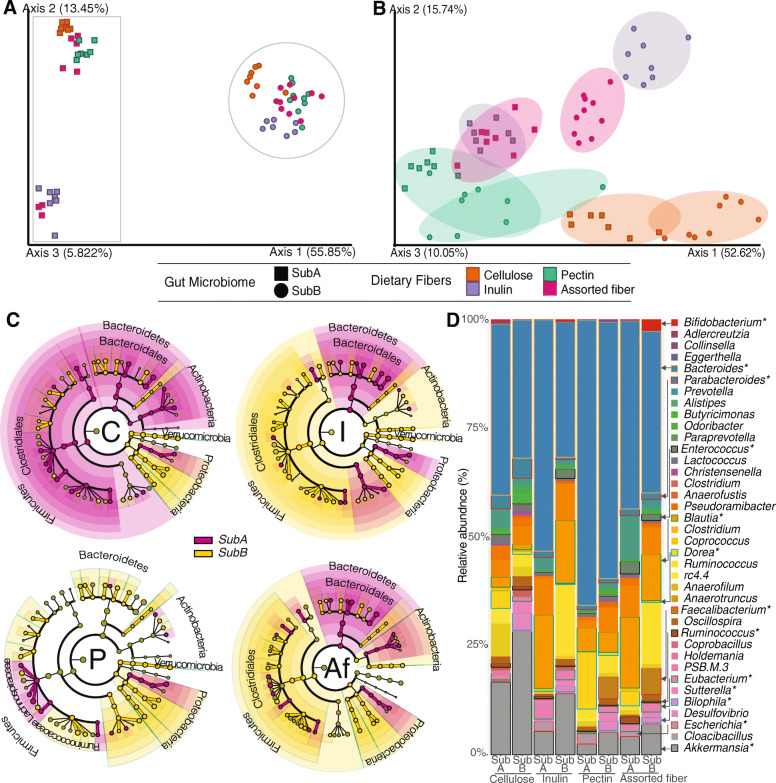
Dietary fibers cause significant restructuring of transplanted human-derived microbial communities. 16S rRNA gene sequence analysis of cecal communities of gnotobiotic mice colonized with SubA and SubB exposed to diets containing cellulose (orange), inulin (purple), pectin (pink), or assorted fiber (green). **a**, **b** Principal coordinates analysis (PCoA) of unweighted and weighted UniFrac distances, respectively. SubA community is represented by squares and SubB by circles. **c** Cladograms generated using LEfSe analysis; comparison results are presented for the two communities in each diet, colors distinguish taxa differences between SubA (magenta) and SubB (yellow) communities. Diet is indicated in each of the four cladograms (C = cellulose, I = inulin, P = pectin, Af = assorted fiber). **d** Genus/family level relative abundances of taxa. Taxa that showed significant differences in relative abundance through the interventions are marked with an asterisk (Kruskal-Wallis test and LDA *P* < 0.05)

16S rRNA gene sequences were generated from cecal samples collected from the mice described above (Tables S3 and S4). PCoA of unweighted UniFrac distances—a metric sensitive to taxonomic phylogenetic distances that does not consider abundance—of these samples show a clear clustering by donor community (Fig. 3a) and less by diet, supporting the notion that all four diets support colonization of similar assemblages for both communities. In all diets, mice colonized with the same donor preserved a core of common species that differentiated it from mice colonized with the other community (Fig. S4A). Nevertheless, there were concomitant subtle richness and pronounced abundance variations within each donor community across the different fiber treatments (Fig. S4). PCoA of weighted UniFrac distances between both communities in the four different diet treatments (Fig. 3b) shows that SubA and SubB post-intervention microbiomes (i.e., microbiome in each diet) cluster separately; however, the inulin diet appeared to be the treatment that separated the two communities the most (Fig. S5). Furthermore, the two communities shifted consistently in response to the same fibers (Fig. 3b), suggesting that related taxa from both communities are responding similarly to a given diet and that the different fibers have distinct effects on abundance of the taxa. PERMANOVA on weighted and unweighted UniFrac distances between engrafted microbiomes derived from the two communities for each dietary fiber intervention showed that these are different in all four diet comparisons (*P <* 0.05). Each diet resulted in a unique set of differences between the two microbial communities and included phyla-level variations. Remarkably, the Firmicutes:Bacteroidetes (F/B) ratio was significantly increased in SubB-colonized mice consuming inulin and assorted fiber relative to SubA-colonized animals in these diets respectively, whereas the F/B ratio was higher in animals colonized with SubA consuming the cellulose diet relative to SubB counterparts, and there was no difference in this ratio between the two communities for mice consuming the pectin diet (Fig. S6A, B).

We used linear discriminant analysis effect size (LEfSe) analysis to identify bacterial taxa significantly contributing to the differences observed between post-intervention microbiomes for each diet [46] (Fig. 3c; Fig. S7). In the cellulose diet, SubA-colonized mice showed higher overall abundance of Firmicutes, Bacteroidetes, and Actinobacteria, whereas in SubB-colonized animals Verrucomicrobia and Proteobacteria exhibited increased abundance. Interestingly, phylum level comparisons between the two communities yielded similar differences for the inulin and assorted fiber-fed mice. Animals colonized with the SubB community showed higher levels of Firmicutes, Actinobacteria, and Proteobacteria, whereas mice colonized with SubA had higher relative abundance of Bacteroidetes in both diets. At the genus level, SubB-colonized animals showed higher relative abundance of *Bifidobacterium* in these two diets, whereas mice harboring the SubA microbiome showed increased levels of *Eubacterium*, *Bacteroides*, *Butyricimonas*, *Lactococcus*, and *Ruminococcus* (Ruminococcaceae) relative to SubB-colonized animals (*P* < 0.05). In the pectin diet, mice colonized with the SubB microbiome showed higher levels of the Proteobacteria phylum and higher levels of the *Akkermansia*, *Faecalibacterium*, *Eubacterium*, and *Clostridium* genera whereas SubA-colonized mice showed higher levels of the Lachnospiraceae family and *Dorea* genus (*P* < 0.05); some of the changes observed in pectin (e.g., *Holdemania*, Lachnospiraceae, and *Eubacterium*) exhibit the opposite pattern seen in mice fed inulin (Figs. S7 and S8).

It is also important to note that some of the genera that showed diet-specific differences between communities were only detected in one of the communities; these included *Bifidobacterium*, *Clostridium*, and *Faecalibacterium*, all detected only in SubB-colonized mice*.* Interestingly, while the bifidogenic effect of inulin has been well documented [47], in our study the assorted fiber diet, which has only 2% inulin, supported significantly higher levels of this genus than the inulin diet, which contains 10% w/w inulin. However, assorted fiber diet also contains scFOS and resistant starch—both known to have bifidogenic effects [48]. It is also possible that the inclusion of additional substrates opens niche opportunities for taxa that would otherwise compete with Bifidobacteria for inulin.

Gas chromatography-based quantification analyses revealed dietary fiber-specific differences in cecal levels of the SCFA acid butyrate and valerate, whereas levels of acetate and propionate were comparable between communities for each diet (Fig. S9). Butyrate was significantly increased in the cecum of SubB-colonized mice consuming cellulose, pectin, and assorted fiber relative to the SubA-colonized animals consuming the same diets (Fig. S9B). Valerate was significantly higher in the gut of SubA-colonized animals in all diets except assorted fiber, whereas cecal levels of the branched-chain fatty acids (BCFA) isovalerate and isobutyrate were higher in SubA-colonized mice relative to SubB counterparts in the cellulose diet (*P* < 0.05; Fig. S9E). Altogether, these results illustrate the divergent effects of dietary fibers on microbial-derived SCFA and BCFA production, which is in consistent with previous work [49, 50].

SubA and SubB were selected based on their distinct abilities to produce butyrate when transplanted to mice fed the assorted fiber diet (Figs. S2 and S9B). While previous work suggests that butyrate has a protective role against metabolic disease [40, 51], comparisons of cecal butyrate levels between mice colonized with the two communities for each diet tested did not fully explain differences in the observed metabolic phenotypes, suggesting that butyrate is not a major contributor to the phenotypic differences observed between SubA- and SubB-colonized mice (Fig. S9B, Fig. 2). However, Pearson correlation analysis across all mice in the study revealed that cecal levels of butyrate were negatively associated with adiposity (*r* = − 0.4682 *P* = 0.003); similar results were seen for cecal levels of acetate (*r* = − 0.3595 *P* = 0.0266) and total SCFA (*r* = − 0.3226 *P* = 0.0483). No significant associations were detected between any of the SCFAs and the other phenotypes measured. These results suggest that while butyrate and potentially acetate may influence adiposity, there are likely other microbial, community-specific, fiber type-dependent metabolites or microbial signals that modulate their effects on this phenotype.

### Post-intervention microbiomes mediate community effects on metabolic outcomes

We sought to evaluate the potential mediating role of post-intervention microbiomes in the SubA/B community effects on metabolic outcomes (adiposity, liver TG, and serum glucose) within each diet group. We focused this analysis on the cellulose, inulin, and pectin dietary fiber interventions, as these included a single type of fiber, as opposed to the assorted fiber which included a mixture of several fibers (RS type 2 and 4, scFOS, inulin, and pectin). We first performed association tests between post-intervention microbiomes (beta-diversity) and host metabolic phenotypes using PERMANOVA. In the inulin diet, post-intervention microbiomes showed differences in beta-diversity associated with changes in liver TG (*P* < 0.05) and glucose phenotypes (*P* < 0.01), whereas differences between the two microbiomes in pectin-fed mice were associated with variation in the adiposity phenotype (*P* < 0.05). We then assessed the mediation effect of the gut microbiome in the relationship between communities and host metabolic phenotypes. The distance-based mediation test showed significant mediation effect of overall diversity of microbiome for the glucose outcome in the inulin-fed group (unweighted UniFrac and Jaccard *P* < 0.05). To identify mediator taxa, we applied a causal mediation model on each internal node of the taxonomy tree (Table S5). For the adiposity outcome, we found the Proteobacteria phylum, the Bacteroidales order, and the Christensenellaceae family as mediators in pectin-fed mice (*P* < 0.05). For the serum glucose outcome, we found Clostridiales order in inulin-fed mice (*P* < 0.05). Altogether, these analyses further support the notion that dietary fiber-microbe interactions modulate metabolic phenotypes and highlight potentially relevant taxa.

### Gut microbiome-dietary fiber interactions modulate blood metabolites

**Fig. 4:**
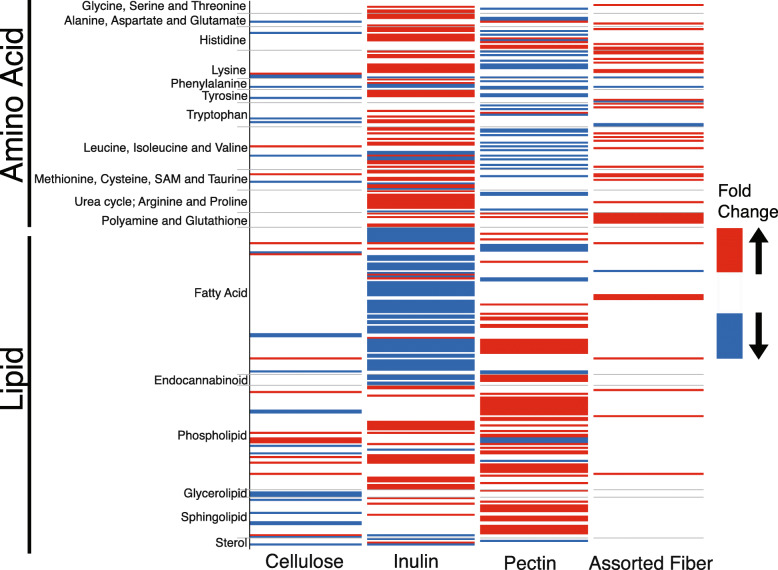
Gut microbiome variation directs changes in serum levels of amino acid and lipid metabolites. Heatmap indicating fold-changes in the abundance of amino acid and lipid metabolites in serum from mice colonized with SubA and SubB communities for each diet as determined by ultrahigh-performance liquid chromatography-tandem mass spectroscopy (UPLC–MS/MS). Biochemicals exhibiting a difference of at least 20% between SubB and SubA are indicated for each diet; *P* < 0.05 (Two-way ANOVA). List of metabolites, *P* values, and fold-changes are listed in Supplemental Table S6

Gut microbes influence host metabolism at least in part by modulating nutrient availability and through the production of a myriad of metabolites, many of which reach systemic circulation [14–17]. We sought to examine whether the gut microbial differences described above between mice colonized with SubA and SubB in the four diets resulted in changes in host metabolism as assessed by metabolomics of serum samples. We applied ultrahigh-performance liquid chromatography-tandem mass spectroscopy (UPLC–MS/MS) to quantify 774 compounds in serum from the 8 groups of mice described above (6 samples/community/diet) (Table S6). Two-way ANOVA analysis between SubB- and SubA-transplanted mice, identified 1 serum metabolite in the cellulose diet, 235 in the inulin diet, 160 in the pectin diet, and 19 in the assorted fiber diet that showed significant differences between the two communities (*P* < 0.05, false discovery rate (FDR) adjusted*-P* < 0.1). A large fraction of these metabolites also showed a significant diet by community interaction (*P* < 0.05; Table S6). For the purpose of generating hypotheses, all metabolites showing *P* < 0.05 were used for further analyses. Animals consuming inulin and pectin diets showed the largest number of significant changes in biochemicals between the two communities. Inspection of the differentially abundant biochemicals within each diet revealed diet-specific changes in metabolites related to amino acids and lipid metabolic pathways, including alanine-aspartate, histidine, lysine, tyrosine, leucine-isoleucine-valine, urea cycle: arginine and proline, methionine-cysteine-taurine, fatty acids, endocannabinoid, and sphingolipid metabolic pathways (Fig. 4, Table S6). These results support the notion that consumption of dietary fiber elicits individual responses on blood metabolites that are influenced by differences in the gut microbiome.

### Amino acids and lipid metabolic pathways associate with host phenotypes

**Fig. 5:**
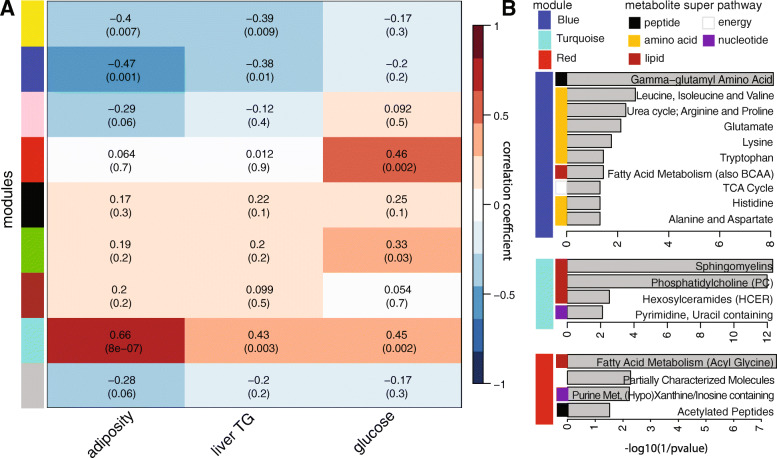
Changes in serum levels of amino acids and lipids associated with metabolic phenotypes. **a** Correlation matrix between metabolite consensus modules and host phenotypes measured in mice described in Fig. 1. Modules were determined based on patterns of co-abundance of metabolites using weighted correlation network analysis (WGCNA). Each of the modules was labelled with a unique color as an identifier. Each module was tested for correlation with host metabolic phenotypes. Within each cell, upper values are correlation coefficients between module and the phenotypes; lower values are the corresponding FDR adjusted*-P* values. **b** Pathways enriched in the blue, turquoise, and red modules as determined by Fisher’s test

We tested whether levels of serum metabolites were associated with host metabolic outcomes. We used the dynamic tree cut method in weighted correlation network analysis (WGCNA, Fig. S10; Table S7) to define modules of tightly correlated serum metabolites. The associations between these modules and host metabolic phenotypes are shown in Fig. 5a. Candidate biochemicals that belong to these enriched pathways are listed in Table S6. The turquoise module is positively correlated with adiposity (*r* = 0.66, *P* = 8e−07), liver TG (*r* = 0.43, *P* = 0.003), and serum glucose (*r* = 0.45, *P* = 0.002), whereas the blue module yielded negative correlations with adiposity (*r* = − 0.47 *P* = 0.001) and liver TG (*r* = − 0.38 *P* = 0.01). The red module showed highest association with the glucose phenotype (*r* = 0.46 *P* = 0.002). Pathway enrichment analysis revealed significant over-representation of metabolites related to sphingomyelins, phosphatidylcholine (PC), and hexosylceramides pathways in the turquoise module whereas the blue module was enriched for metabolites in the gama-glutamyl amino acid, branched-chain amino acid (BCAA), urea cycle, glutamate, lysine and tryptophan pathways and the red module in fatty acid metabolism (acyl glycine), purine metabolism ((Hypo)Xanthine/Inosine containing), and acetylated peptides (Fig. 5b).

Sphingomyelins (turquoise module) are strongly associated with adiposity, TG, and glucose levels. These metabolites are increased in SubB-colonized mice in the pectin diet, which exhibited increased adiposity relative to SubA-colonized mice. Previous work indicates that sphingolipids mediate cellular processes involved in apoptosis, cell differentiation, and inflammation [52]. Furthermore, serum levels of sphingomyelins and ceramides have been associated with the development of obesity [53]. Sphingomyelins can be hydrolyzed by sphingomyelinases releasing phosphocholines and ceramides, leading to metabolic impairment [53]. Furthermore, most of the identified sphingomyelin-lipid metabolism changes are within the pectin-fed group (Tables S6 and S8). These results support the notion that microbiome-fiber interactions modulate host levels of sphingolipids and ceramides. This is consistent with recent work suggesting that *Bacteroides*-derived sphingolipids in the intestine provide an endogenous source of sphingolipids to the host [54].

As mentioned above, amino acids are enriched in the blue module and largely contribute to the negative association observed between this module and adiposity, liver TG, and glucose levels. These include histidine, which was increased in SubB-colonized mice consuming the inulin diet compared to SubA-colonized counterparts (Table S8). SubB-colonized animals in this diet exhibited lower glucose and adiposity relative to the SubA-colonized animals (Fig. 2). This is consistent with previous work showing that histidine supplementation results in an improvement in insulin sensitivity and lower body fat [55]. Several bacterial metabolites derived from histidine were also increased in SubB-colonized mice in the inulin diet compared to SubA-colonized animals, including imidazole propionate (Table S6), which has been linked to impaired insulin signaling and type II diabetes in humans [15, 56].

BCAAs were also enriched in the blue module and detected at higher levels in SubB-colonized mice in the inulin diet compared to SubA-colonized animals. Previous reports indicated that BCAAs upregulate glucose transporters and activate insulin secretion [57, 58]. However, there is also evidence that leucine and isoleucine have a negative impact on metabolic health [59, 60], and several studies have suggested that excessive intake of amino acids could lead to inhibition of insulin signaling [61]. Additionally, the bacterial metabolite derived of tryptophan, indolepropionate [62], which we detected at significantly higher levels in mice colonized with the SubB community consuming inulin relative to SubA counterparts, has been previously associated with increased dietary fiber intake and linked to reduced risk of low-grade inflammation [63] and improved glucose homeostasis [64]. Mice fed the cellulose diet showed virtually no differences in the metabolites enriched in this module. Gamma-glutamyl amino acids were also highly enriched in the blue module. Gamma-glutamyl dipeptides are also involved in glutathione (GSH) metabolism, which plays an important role in antioxidant defense, and are produced when gamma-glutamyl transpeptidase catalyzes the transfer of the gamma-glutamyl moiety of glutathione to amino acids. Gamma-glutamyl transpeptidase is expressed in several mammalian tissues and in bacteria [65, 66]. While the role of these metabolites on metabolic health remains poorly understood, a recent study showed gamma-glutamyl amino acids γ-glutamyl cysteine and γ-glutamyl valine inhibit TNF-α signaling in intestinal epithelial cells and reduce inflammation [67]. Overall, these results suggest that circulating levels of sphingolipids, amino acids, and gamma-glutamyl dipeptides are impacted by interactions between microbes and dietary fibers. Interestingly, these circulating metabolites also showed significant correlations with cecal levels of acetate and butyrate (Fig. S11). While further studies are needed to assess the role of these two SCFAs in modulating the abundance of systemic metabolites, both acetate and butyrate are known to impact many facets of metabolism via interactions with G-protein coupled receptors present in the gut and in the periphery [20].

### Connecting bacterial taxa, serum metabolites, and host metabolic phenotypes

**Fig. 6:**
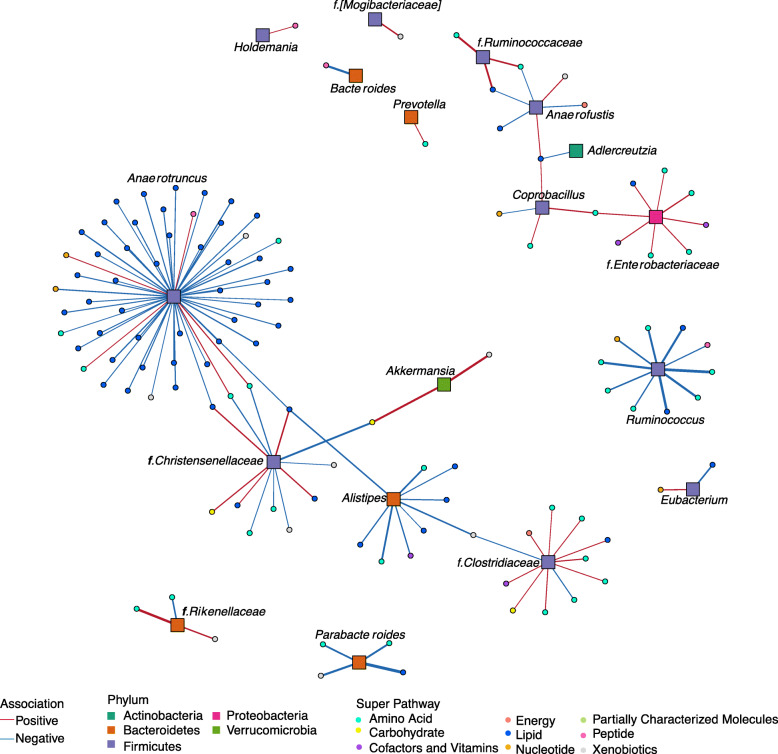
Association network between gut microbiota and blood metabolites. Association strength is denoted by width of the lines; red lines show positive association while blue ones show negative. Phylum of taxa is indicated by colored boxes and metabolite super-pathway (listed in Supplemental Table 5) by colored circles. *Anaerotruncus* (Firmicutes) is the genus with the largest number of negative associations with the lipid super-pathway, while the family Chistensenellaceae (Firmicutes) has the largest number of positive associations with metabolites in the same super-pathway. Enterobacteriaceae family (Proteobacteria) is the taxa with more positive associations with the amino acid super-pathway

To identify bacterial taxa associated with metabolites, we applied a log-contrast model with metabolites as the response. The association analysis linking taxa and metabolite super-pathways show that the Firmicutes phylum is involved in most associations, followed by Bacteroidetes (Fig. 6). Interestingly, the genus *Anaerotruncus* is negatively associated with metabolites of the lipid super-pathway that includes fatty acids (long-chain saturated and unsaturated, and branched), lysophospholipids, and monacylglycerol. This genus is also positively associated with metabolites of the amino acid and the nucleotide super-pathways, including lysine, glycine, arginine-proline metabolism, and the purine and pyrimidine. *Ruminococcus* (Ruminococcaceae) is negatively associated with metabolites of the amino acid and the lipid super-pathways, including BCAA, glutamate and tryptophan metabolism, fatty acids, purine, and gamma-glutamyl amino acid, whereas *Parabacteroides* is negatively associated with metabolites in the arginine-proline and dihydroxy fatty acid pathways. Interestingly, *Anaerotruncus* and *Ruminococcus* (Ruminococcaceae), along with *Parabacteroides*, showed significantly higher relative abundance in mice colonized with the SubA community in the inulin diet, which exhibit higher adiposity, liver TG, and glucose compared to mice in the same diet colonized with the SubB community (*P* < 0.01; Fig. S8).

The Rikenellaceae family showed a strong positive association with tryptophan and tyrosine metabolism and a negative association with BCAA metabolism. Interestingly, Rikenellaceae and Ruminococcaceae were also positively correlated with cecal levels of BCFAs—the end products of bacterial BCAA catabolism (Fig. S12). The relative abundance of Rikenellaceae was significantly increased in SubA-colonized mice consuming cellulose, inulin, and assorted fiber and in SubB-colonized mice consuming pectin, which exhibited worse metabolic outcomes relative to the other community in their respective diets. This is consistent with previous reports describing increased abundance of Rikenellaceae in leptin-resistant obese and diabetic mice [68, 69]. Moreover, we found this family was positively associated with genes in the glycerophospholipid metabolic process, which generates lipids that can be packed in very low-density lipoproteins [70]. The *Bacteroides* genus showed a strong negative correlation with gamma-glutamyl amino acids, and it was detected at significantly higher levels in SubA-colonized animals except when animals were fed pectin (*P* < 0.05). *Eubacterium* was negatively associated with plasmalogens and positively associated to purine metabolism. As discussed above, this genus was also more abundant in SubA-colonized mice consuming inulin diet relative to SubB-colonized counterparts, and in SubB-colonized mice consuming pectin diet relative to SubA-colonized animals in the same diet. In both cases, the increased levels of *Eubacterium* were associated with less favorable metabolic outcomes (Fig. 2 and Fig. S8). Altogether, these results link changes in levels of bacterial taxa differentially represented in the two communities across the different diets with alterations in systemic levels of metabolites and host metabolic phenotypes.

### Dietary modulation of gut microbiome influences impact of gut communities on hepatic gene expression

**Fig. 7:**
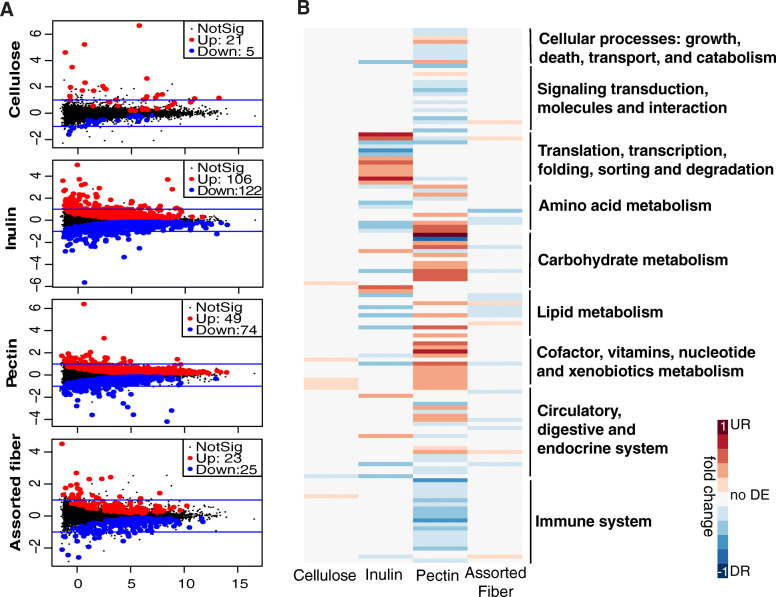
Effects of gut microbiome variation on hepatic gene expression across different fibers. **a** MA plots showing differentially expressed genes in the liver of mice colonized with SubB *vs.* SubA microbiome consuming (i) cellulose diet; (ii) inulin diet; (iii) pectin diet; (iv) assorted fiber diet (*n* = 5 animals/microbiome/diet). Differentially express genes (DEGs) *P* < 0.05, FDR adjusted*-P* < 0.05. **b** Heatmap showing differentially expressed KEGG pathways between SubB and SubA. The ratio of significantly regulated KEGG terms and genes annotated with each KEGG term (minimum of 10) comparing SubB *vs*. SubA in each dietary intervention are shown in the heatmap. Upregulated pathways are shown in red and downregulated pathways are shown in blue. Inulin and pectin comparisons show the largest number of differentially expressed genes. MA stands for the relationship between values of intensity (i.e., counts) and difference between the data (M = log ratio and A = mean average)

The liver receives a large fraction of its blood supply through the portal circulation, which is the direct venous outflow of the intestine. As such, the liver is continuously exposed to gut microbial-derived products, including SCFAs and bacterial toxins [71]. Differences in abundance of microbial metabolites, including SCFAs, have been linked to changes in global host epigenetic states and gene expression [72]. Epigenetic states of chromatin are reflected in the covalent post-translational modifications (PTMs) on histone proteins. We found that colonization of mice with SubA and SubB communities affected covalent post-translational modifications (PTMs) on histone proteins (Supporting information, Figs. S13 and S14). To test directly whether these differences were linked to changes in hepatic gene expression, we performed RNA sequencing (RNA-seq) analysis. Tests of differential gene expression yielded larger number of significantly regulated genes between the two communities for the inulin and pectin dietary interventions (Fig. 7), while cellulose-fed mice showed the least number of differences. Only 26 genes were differentially expressed between the two communities in cellulose-fed mice, compared to 228 genes in inulin, 123 in pectin, and 48 in the assorted fiber diets (*P* < 0.05 and FDR adjusted*-P* < 0.05; Fig. 7a, Table S9).

Linear model fit test for over-representation of gene ontology (GO) among differentially expressed genes in biological process (BP), molecular function (MF), and cellular component (CC) categories as well as enrichment analysis on Kyoto Encyclopedia of Genes and Genomes (KEGG) pathways revealed divergent results between dietary interventions (Table S10). Among the transcripts expressed at lower levels in SubB-relative to SubA-colonized mice in the cellulose diet, there were genes associated with regulation of phosphorylation and protein modification. SubB-colonized animals consuming inulin diet showed lower levels of expression of genes associated with amino acid metabolism, fatty acid metabolic process, peroxisome components, oxidoreductase activity, and peroxisome proliferator-activated receptor (PPAR) signaling pathway relative SubA-colonized counterparts. Upregulated genes in SubB-colonized mice were associated with ribosome biogenesis, RNA metabolic process, and protein processing in endoplasmic reticulum. Mice consuming assorted fiber diet showed some similarities in the enrichment of differentially expressed genes as those in the inulin diet. Genes downregulated in SubB-relative to SubA-colonized mice fed inulin and assorted fiber diets showed overlapping GO terms including oxidative stress, regulation of cellular ketone metabolic process, lipid metabolic process, fatty acid metabolic-catabolic process and lipid modification, fatty acid oxidation, long-chain fatty acid metabolic process, and oxidoreductase activity.

In the pectin diet, mice colonized with SubB expressed higher levels of genes involved in cofactor, vitamins, and nucleotide metabolism; fatty acid metabolism, oxidoreductase activity, carbohydrate and valine, leucine and isoleucine metabolism (Fig. 7b, Table S10). These include six genes encoding cytochrome P450, some of which are known to play important roles in the synthesis of steroid hormones (*Cyp2a4, Cyp17a1*) and xenobiotic metabolism (*Cyp2a5*) [73, 74]. Surprisingly, SubA-colonized animals in the pectin diet—which show lower levels of adiposity (Fig. 2a) relative to SuB-colonized mice—exhibited higher levels of expression of genes involved in immune system process, inflammatory response, phagocytic cup and vesicle cellular component, cytokine receptor activity and binding, and infection pathways. Previous work has shown that the overall health effects of consumption of MACs such as inulin and pectin can be context dependent. Long-term consumption of pectin or inulin by Toll-like receptor 5 (TLR5) KO mice, which show innate immune deficiencies and gut microbiome alterations, results in liver inflammation and hepatocellular carcinoma [75]. Altogether, these results suggested that the gut microbiome is a differential factor that modulates hepatic gene expression. Furthermore, data suggests that the impact of the gut microbiome on liver gene expression is influenced by the type of dietary fiber consumed.

### Liver gene expression is associated with host metabolic phenotypes

**Fig. 8:**
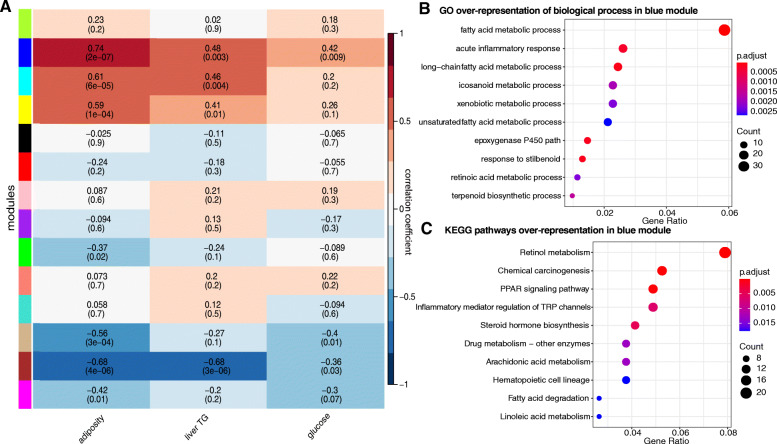
Liver gene expression is associated with host metabolic phenotypes. **a** Correlation matrix between gene expression consensus modules and host phenotypes measured in mice described in Fig. 1. Modules were determined based on patterns of co-abundance of transcripts using weighted correlation network analysis (WGCNA). Each of the modules was labelled with a unique color as an identifier. Each module was tested for correlation with host metabolic phenotypes. Within each cell, upper values are correlation coefficients between module and the phenotypes; lower values are the corresponding FDR adjusted*-P* values. **b** Gene Ontology (biological process term) and KEGG pathways over-represented in the blue module (gene count > 5; *P* and FDR adjusted*-P* < 0.05)

WGCNA was applied to the normalized read count data obtained from RNA-Seq analysis. Fourteen gene modules, each clustering highly co-expressed genes, were identified (each module was assigned a different color; Fig. S15, Table S11). We performed correlation analysis between the phenotypic data and the calculated eigengene—defined as the first principal component of the expression matrix of the corresponding module—for each module identified (Fig. 8a). The blue module and adiposity showed the strongest association (*r* = 0.74, *P* = 2e−07). We narrowed down possible relevant genes contributing to this module by performing biological process GO and KEGG pathway analysis enrichment (Fig. 8b, c). In total, 614 out of the 677 transcripts in the blue module mapped into biological process gene ontology. These showed significant enrichment in several processes including the fatty acid metabolic process, acute inflammatory response, long-chain fatty acid, and unsaturated fatty acid metabolic process (*P* < 0.05 and FDR adjusted-*P* < 0.05). Pathway enrichment analysis of 266 genes with KEGG annotation in this module yielded significant enrichment in pathways that included PPAR signaling, inflammatory mediator regulation of transient receptor potential (TRP) channels, fatty acid degradation, and linoleic acid metabolism (*P* < 0.05 and FDR adjusted-*P* < 0.05).

When only considering genes in the blue module with significant positive association to each phenotype, we observed 209, 151, and 107 genes for adiposity, liver TG, and glucose, respectively (*P* < 0.05). The GO biological process analysis for this set of genes in the blue module showed that throughout the measured phenotypes, the fatty acid metabolic process was the gene ontology with more gene counts, followed by long-chain fatty acid metabolic process, xenobiotic metabolic process, and epoxygenase P450 pathway. The statistically significant (*P* < 0.05 and FDR adjusted-*P* < 0.05) enriched KEGG pathways associated with the measured phenotypes were retinol metabolism, chemical carcinogenesis, PPAR signaling pathway, steroid hormone biosynthesis, linoleic acid metabolism, biosynthesis of unsaturated fatty acids, and fatty acid elongation. Nucleoside, ribonucleoside and purine nucleoside bisphosphate metabolic process, and fatty acid degradation are the GO terms and KEGG pathways that were enriched in the association with adiposity and liver TG (Fig. S16), whereas inflammatory mediator regulation of TRP channels and pyruvate metabolism pathways are enriched among the genes associated with glucose levels.

### Gene expression in liver is associated with abundance of gut bacterial taxa

**Fig. 9:**
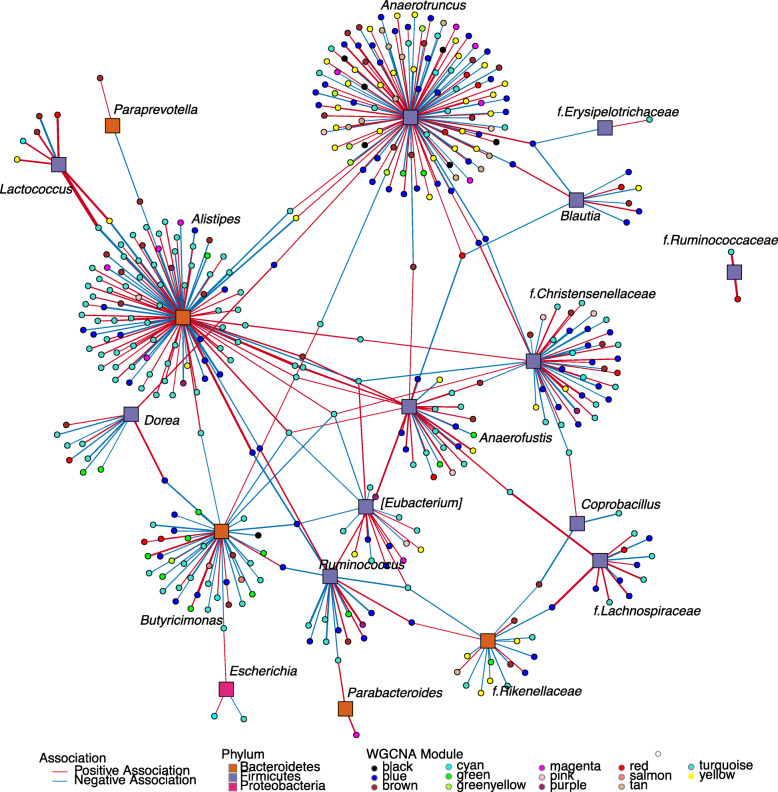
Taxa associated with liver transcriptomic modules. Association strength is denoted by width of the lines, red lines show positive association while blue ones show negative. Phylum level classification of the taxa is marked by colored boxes and transcriptomic weighted correlation network analysis (WGCNA) module by colored circles. *Anaerotruncus* (Firmicutes) and *Alistipes* (Bacteroidetes) are the taxa with the most associations with the clustered genes

The association analysis linking taxa and liver gene expression modules showed that the Firmicutes phylum is involved in most associations, followed by Bacteroidetes (Fig. 9). Interestingly, *Anaerotruncus* cluster has the highest number of gene associations (334), followed by *Alistipes* (105), *Butyricimonas* (58), and Christensenellaceae (49) (Table S12). This association network revealed an overall positive association between *Anaerotruncus* with genes belonging to the lipid metabolic process, and a negative association with genes belonging to purine ribonucleotide and nucleoside metabolic process, cholesterol biosynthetic process, cellular lipid, fatty acid and phospholipid biosynthetic process, and response to cytokines. Interestingly, serum metabolomics data shows an overall negative association of this genus with metabolites of the lipid super-pathway (Fig. 6), suggesting that these taxa may play a role in host lipid metabolism. *Alistipes* was positively associated with genes in the lipoprotein biosynthetic process clustered in the turquoise module, whereas *Butyricimonas* was overall negatively associated with immune system processes. Previous work has found the *Anaerotruncus* and *Alistipes* genera enriched in genetically obese (*ob/ob*) mice exhibiting severe glycolipid metabolism disorders relative to wild type and *ob/ob* mice consuming inulin with improved metabolic parameters [76, 77]. Abundance of these two genera was also linked with consumption of high-fat diets in humans [78–80]. Altogether, these studies suggest that *Anaerotruncus* and *Alistipes* may directly or indirectly contribute to the metabolism of dietary fats and fiber types and community context influence this dynamic. Furthermore, the strong correlations between intestinal microbes, hepatic gene expression, and host metabolic phenotypes provide potential connections between dietary fiber-microbes-health that warrant further examination.

## Conclusion

Human and mouse studies encompassing genetically diverse populations have shown that host’s genetic variation impacts all facets of physiology including responses to diet [81]. However, it is now clear that these populations also contain a significant amount of genetic variation derived from their largely individual associated microbiomes. Previous work in humans showed that dietary supplementation of resistant starch increases fecal butyrate levels, but with remarkable interindividual variation [50]. Furthermore, a recent study suggested that individual gut microbiota differences can be used to predict post-prandial glycemic responses to specific foods [82]. While these studies provide strong support to the notion that the gut microbiome is a major source of variability that influences responses to diet, dissecting the effects of microbial *vs.* host genetic variation while controlling environmental exposure is practically impossible to achieve in human studies. Modeling this variation in GF mice provides the opportunity to unravel the effects of host genetics from environmental and microbial exposures allowing the discovery of causal relationships between microbes and host phenotypes [76, 77]. Using this approach, we demonstrate that consumption of the same dietary fiber by genetically identical mice harboring different human-derived microbiomes can lead to different host metabolic outcomes (Fig. 2). Remarkably, these phenotypic responses varied as a function of the type of fiber present in the diet and were associated with changes in cecal and blood metabolites, and hepatic gene expression.

A recent study using antibiotics and PEG treatment to deplete the mouse indigenous gut microbiota, followed by transplant of human fecal microbiota from four obese donors into these animals which were fed a high-fat diet, showed that host metabolic effects in response to inulin supplementation varied as a function of the human community used to colonize the mice [83]. This work also identified several genera including *Barnesiella*, *Bilophila*, *Butyricimonas,* and several *Alistipes* amplicon sequence variants (ASVs) positively correlated with adiposity and/or hepatic steatosis whereas *Akkermansia*, *Raoultella*, and *Blautia* were negatively correlated with at least one of these metabolic outcomes in the transplanted mice. Some of these findings are consistent with our results, e.g., *Alistipes* and *Butyricimonas* were detected at lower levels in SubB-colonized subjects in the inulin diet relative to SubA-colonized mice in the same diet, whereas *Akkermansia* was detected at higher levels in SubB-colonized in this diet, which showed an overall healthier metabolic profile than SubA-colonized counterparts. Interestingly, higher basal levels of *Akkermansia* were detected in the gut microbiomes of obese individuals that benefited from a dietary inulin intervention, (i.e., responders) relative to non-responders of the same treatment [83]. Furthermore, higher baseline levels of *Akkermansia* were also associated with better clinical outcomes among individuals with metabolic syndrome subjected to a calorie-restricted diet [84]. Further studies are needed to establish the causal relationship between *Akkermansia* and enhanced response to this MAC and to identify potential molecular/microbial players involved.

The results presented here, combined with the work discussed above, suggest that the efficacy of dietary interventions such as prebiotics depends on the gut microbiota of the consumer and one-fits-all approaches to promote health are unlikely to elicit consistent effects across individuals. Identifying gut microbial biomarkers associated with beneficial responses to common interventions may help to stratify subjects into more effective personalized treatments.

While ascertaining causal mechanisms explaining differences in host metabolic phenotypes between microbiomes for each diet is beyond the goal of the current study, our results illustrate how introduction of MACs promote divergent host phenotypes caused by the two gut communities. This result has implications not only for personalized nutrition approaches but is also relevant for animal studies in the nutrition field. The use of AIN93-based purified diets [85] that contain cellulose as the sole source of fiber is common practice in nutrition studies. These diets are useful tools for the field, but their lack/low levels of dietary MACs might not support gut bacterial taxa that are relevant to the process studied. While there is not sufficient evidence to prescribe the use of a particular fiber or a specific combination of different fibers, the presented results describe some of the consequences of their inclusion or lack thereof. Furthermore, inclusion of some fermentable fibers in rodent diets will enable conditions that are more representative of human and rodent nutrition.

A major limitation of the study is that only two communities were compared. While these showed clear organismal and functional differences (Figs. S1 and S3) and elicited distinct host metabolic phenotypes, they likely capture a small fraction of the variability observed among human gut microbiomes. Expanding this study to include a wider range of communities, including samples from subjects with extreme diets, markedly different lifestyles, or different health status may expand the range of responses to dietary fiber mediated by gut microbes. Additionally, our study did not characterize the degree to which each of the fibers was metabolized by the two communities. Identifying gut signatures associated with desired health outcomes in response to specific fibers may reveal biomarkers of beneficial diet-microbiome interactions that guide personalized nutrition approaches. In conclusion, the present study underscores the importance of the gut microbiome as a differential factor that contributes to individual variation in metabolic responses to dietary fiber.

## Methods

### Dietary formulation

The four diets used during this study contained 35% kcal fat, 20% kcal protein, 45% kcal carbohydrate, and different fiber type which equals 10% weight. A non-fermentable fiber, cellulose (Solka Floc), was used as control while inulin [Oliggo-Fiber Inulin instant (100010911); Cargill, Minneapolis, MN], pectin (PE1006; Gojira Fine Chemicals, LLC, Bedford Height, OH), and a formulation of assorted fibers which contained 23.4 g/kg inulin, 21.5 g/kg short-chain fructooligosaccharide (i.e., scFOS, NUTRAFLORA®, Ingredion Inc., Westchester, IL), 33.3 g/kg resistant starch type 2 (HI-MAIZE® 260, Ingredion Inc., Westchester, IL), 23.5 g/kg resistant starch type 4 (Fibersym®, MPG, Atchison, KS), 23.5 pectin, as sources of fermentable fibers (Table S1). Experimental diets were manufactured and sterilized via irradiation by Envigo (10% cellulose diet; TD.170720, 10% inulin diet; TD.170721, 10% pectin diet; TD.170725, and assorted fiber diet; TD.170726).

### Gnotobiotic husbandry

All experiments involving gnotobiotic mice were performed under protocols approved by the University of Wisconsin-Madison Animal Care and Use Committee. All germ-free (GF) C57BL/6 mice were maintained in a controlled environment in plastic flexible film gnotobiotic isolators under a strict 12-h light/dark cycle and received sterilized water and chow (LabDiet 5021; LabDiet, St. Louis, MO) ad libitum. A week prior to colonization, mice were switched to the assorted fiber diet. GF status of mice was confirmed prior to starting the experiment using culture-dependent methods. No turbidity was observed when feces collected from the mice were inoculated in a panel of rich media and incubated at 37 °C aerobically and anaerobically for a week.

### Colonization of germ-free mice and dietary fiber interventions

#### Screening phase protocol

Richness and diversity metrics (i.e., alpha and beta) obtained from previous publication [38] plus Faith’s phylogenetic diversity (calculated using QIIME 2 [86]) were used to describe 8 Wisconsin Longitudinal Study (WLS) samples selected for this study [87]. All donors were adults between 70 and 82 years old, (6 men, 2 women) with reported body mass index interval was between 23 and 38. Fecal suspensions were prepared under anaerobic conditions in Hungate tubes. A ~ 0.5-cm piece of frozen fecal aliquot straw technique (FAST) straw material were resuspended in 5 ml mega media as previously described [16, 88]. Adult 6–8-week-old male C57BL/6 GF mice (total *n* = 23) were inoculated by oral gavage with ~ 200 μl of fecal suspension (*n* = 2–4 mice/sample), after being fed with an assorted fiber diet (see below) for a week in sealed positive pressure individually ventilated cages (IVCs; Allentown). Each cage contained 2–4 mice. Upon inoculation, mice were maintained on the same assorted fiber diet for 2 more weeks. Cecal contents were collected for SCFAs and 16S rRNA gene sequencing analysis from each of the 8 gnotobiotic groups.

#### Study design

The two fecal communities exhibiting the highest (SubB) vs. lowest (SubA) butyrate-producing activity were then selected for subsequent experiments. Both samples belonged to individuals of the same sex (males), similar age (76.5 ± 1 years old), BMI = 30, and with no history of diabetes, cancer, or heart disease. Three-day diet recall indicates that both subjects consumed a typical western diet. Fecal samples from SubA and SubB were inoculated into 7–9-week-old GF male mice (*n* = 30–36 mice/community) consuming assorted fiber diet as described above. All gnotobiotic mice continued with the same irradiated assorted fiber diet for two more weeks (stabilization period). Bedding and wires with food were exchanged between cages of mice colonized with the same community to minimize cage effects. Mice were then switched to one of the four diets described above: cellulose, inulin, pectin, and assorted fiber (*n* = 7–10 mice/community/diet), and maintained in these diets for another 4 weeks. Animals were euthanized after 4 h of fasting.

### Measurements of short-chain fatty acids (SCFA)

#### SCFA analysis of mouse samples

Cecal levels of SCFAs were measured as previously described [19]. Briefly, a mixture of 10 μl of internal standards (200 mM for mice and 20 mM for human each; acetic acid-D4, Sigma-Aldrich no. 233315; propionic acid-D6, Sigma-Aldrich no. 490644; and butyric acid-D7, CDN isotopes no. D-171) was subsequently added, followed by 20 μl of 33% HCl and 1 ml diethyl ether. The vials were sealed, vortexed vigorously for 3 min, and then centrifuged (4000*g*, 10 min). The upper organic layer was transferred to another vial and a second diethyl ether extraction was performed. After combining the 2 ether extracts, a 60 μl-aliquot was removed, combined with 2 μl *N*-tert-butyldimethylsilyl-*N*-methyltrifluoroacetamide (Sigma-Aldrich no. 394882) in a GC auto-sampler vial with a 200 μl glass insert, and incubated for 2 h at room temperature. Derivatized samples (1 μl) were injected onto an Agilent 7890B/5977A GC/MSD instrument with an Agilent DB1-ms 0.25 mm × 60 m column with a 0.25-μm bonded phase. A discontinuous oven program was used starting at 40 °C for 2.25 min, then ramping at 20 °C min^−1^ to 200 °C, then ramping at 100 °C min^−1^ to 300 °C and holding for 7 min. The total run time was 18.25 min. Linear column flow was maintained at 1.26 ml min^−1^. The inlet temperature was set to 250 °C with an injection split ratio of 15:1. Acquisition B.07.02.1938. The m/z values of monitored ions in mice cecal measurements were as follows: 117 (acetic acid), 120 (acetic acid-D4), 131 (propionic acid), 136 (propionic acid-D6), 145 (butyric acid), and 152 (butyric acid-D7). Concentrations were normalized to milligrams of cecal contents.

### Measurements of branched-chain fatty acids (BCFA)

Levels of BCFA were quantified in cecal samples collected from mice exposed to the four different diets used in the study (*n* = 66; *n* = 7–10 per community/dietary fiber intervention) using the headspace GC analysis method. Frozen, weighed samples (~ 70 mg) were added to chilled 20-ml headspace vials (Restek, Bellefonte, PA) containing 2.0 g NaHSO4, distilled water (300 μl—sample weight), and 1.0 ml of 60 μM 2-butanol (internal standard; added just prior to the sample). Vials were crimp sealed immediately after sample addition and vortexed periodically to disperse and mix the contents. Headspace GC analyses were performed using a Shimadzu (Columbia, MD) HS-20 headspace sampler connected to a Shimadzu GC-2010 Plus GC equipped with a SH-Stabilwax column (30 m, 0.25 mm ID, 0.10 μm df) linked to a FID. Samples were equilibrated with shaking to 80 °C for 20 min, pressurized to 80 kPa for 3 min prior to column injection (2 ml injection loop, load time 0.2 min, sample and transfer line temperature 150 °C, 1:15 split ratio, N2 column flow 1.2 ml/min), with a column temperature program starting at 40 °C/2 min, increased to 200 °C (20 °C/min), held 2 min, decreased to 120 °C (20 °C/min), decreased to 40 °C (40 °C/min), and stabilized 1 min prior to the subsequent injection. The GC cycle time was approximately 23 min. Standard mixtures were prepared and analyzed by the same method, and peak areas determined using Shimadzu Lab Solution software (version 5.92), with adjustment for fecal sample size.

### Tissue collection and analysis

Blood was collected via cardiac puncture of anesthetized mice following a 4-h fast. Serum was obtained by centrifugation and stored at − 80 °C. Cecal contents, liver, and gonadal fat pads were collected at the time of euthanasia, snap frozen in liquid nitrogen, and stored at − 80 °C until analysis. Glucose was measured using ultrahigh-performance liquid chromatography-tandem mass spectroscopy (UPLC–MS/MS) by Metabolon.

### Liver triglyceride measurements

Liver triglycerides (TG) were quantified as previously described [19, 89]. Briefly, between 30 and 40 mg of frozen liver tissue was homogenized in 30 ml of 2:1 chloroform:methanol and disrupted using a bead beater (BioSpec Products, Barlesville, OK; maximum setting for 6 min at room temperature). Samples were incubated overnight at 4 °C with gentle agitation and 1 ml of 4 mM MgCl was added for phase separation. The organic solvent (500 μl) was left to evaporate overnight and the dried lipids were reconstituted in 200 μl butanol:triton- × 114 mix (3:2 vol:vol). TG content was determined by colorimetric assay from Sigma (Sigma, F6428), according to the manufacturer’s instructions and expressed in nanomole per gram of wet tissue for final concentration.

### Statistical analysis of mouse phenotypes

To assess differences on metabolic phenotypes measured between microbiota communities within each dietary intervention and between the same microbiota across different diets, we performed a nonparametric test and use permutation approach to obtain the *P* value for two-group comparison through Wilcoxon rank sum test. Homogeneity of variance was tested using Levene’s test (*P* > 0.05) previous to performing a two-way ANOVA to investigate the effect of dietary intervention, transplanted microbiota community, and their interaction on each phenotype (*P* < 0.05).

### 16S rRNA gene sequencing

Genomic DNA was extracted from cecal contents using a bead-beating protocol [76]. Briefly, ~ 50 mg of fecal pellet sample were resuspended in a solution containing 500 μl of extraction buffer [200 mM Tris:HCl (pH 8.0), 200 mM NaCL, 20 mM EDTA], 210 μl of 20% SDS, 500 μl phenol:chloroform:isoamyl alcohol (pH 7.9, 25:24:1) (Invitrogen 15593-049), and 500 μl of 0.1-mm diameter zirconia/silica beads. Samples were mechanically disrupted using a bead beater (BioSpec Products, Barlesville, OK; maximum setting for 3 min at room temperature), followed by centrifugation, recovery of the aqueous phase with 60 μl 3 M NaAcetate, and precipitation with isopropanol. QIAquick 96-well PCR Purification Kit was used to remove contaminants. Isolated DNA was eluted in 10 mM Tris (pH 8.0) buffer and was stored at − 20 °C until further use.

Amplification of 16S rRNA genes (V4) was done from DNA by PCR using unique 8-bp barcodes on the forward and reverse primers and fused with Illumina sequencing adapters [90]. Each sample was amplified in duplicate in a reaction volume of 12.5 μl using KAPA HiFi HotStart DNA polymerase (KAPA Biosystems, Wilmington, MA, cat. # KK2602), 10 μM of each primer, and ~ 12.5 ng of genomic DNA. PCR was carried out under the following conditions: initial denaturation for 3 min at 95 °C, followed by 25 cycles of denaturation for 30 s at 95 °C, annealing for 30 s at 55 °C and elongation for 30 s at 72 °C, and a final elongation step for 5 min at 72 °C. PCR products were purified with the QIAquick 96-well PCR Purification Kit and then quantified using Qubit dsDNA BR Assay kit (Invitrogen, Oregon, USA). Samples were equimolar pooled and sequenced on the Illumina MiSeq 2 × 250 bp platform.

Sequences were processed using QIIME 2 pipeline [86]. Demultiplexed 250 bases paired-end sequences were imported using Casava 1.8 format and denoised using DADA2 [91, 92] to obtain amplicon sequence variant (ASV) table. Singletons (ASV present < 2 times) and ASVs that are present in less than 10% of the samples were discarded. Greengenes [93] reference sequences (clustered at 99% similarity) were used to train a naïve Bayes taxonomy classifier to further annotate ASVs taxonomically. ASVs were then collapsed based on genus or lowest-level (i.e., family, order, class, phylum) taxonomy possible. An even sampling depth of 5795 and 33,714 sequences per sample was used for assessing alpha- and beta-diversity measures in the screening and study phase, respectively. Shannon diversity Index and Faith’s phylogenetic diversity (PD) was used to measure alpha diversity. Beta-diversity was calculated using principal coordinates analysis (PCoA), Jaccard, and weighted and unweighted UniFrac metrics [94]. Weighted UniFrac distances between microbiota communities were tested by pairwise PERMANOVA using Qiime2 beta-group-significance command with the -p-pairwise parameter [95]. Also, linear discriminant analysis (LDA) effect size (LEfSe Galaxy Version 1.0) was performed to each microbiota pair (SubA and SubB) for each dietary fiber intervention to elucidate significantly different abundances of bacterial taxa. The parameters used for these analyses were set with default *P* value (*α* = 0.05) and LDA score of 2.0 [46].

PICRUSt2 was used to predict functional content or microbiome 16S rRNA genes [43, 44] using QIIME2 generated data. An even sampling depth of 3,919,286 gene counts was used to rarefy all samples to further analyze diversity using Bray Curtis analysis.

### Untargeted metabolomics of serum samples

Untargeted mass spectrometry data was collected at Metabolon Inc from 100 μl serum samples of 6 randomly selected mice in each treatment. The 48 samples were prepared using the automated MicroLab STAR system (Hamilton Company). Recovery standards were added and protein, dissociate small molecules bound to protein or trapped in the precipitated protein matrix were removed. To recover chemically diverse metabolites, proteins were precipitated with methanol under vigorous shaking for 2 min (Glen Mills GenoGrinder 2000) followed by centrifugation. The resulting extract was divided into five fractions: two for analysis by two separate reverse-phase UPLC–MS/MS methods with positive ion mode electrospray ionization (ESI), one for analysis by reverse-phase UPLC–MS/MS with negative ion mode ESI, one for analysis by HILIC/UPLC–MS/MS with negative ion mode ESI, and one sample was reserved for backup. Samples were placed briefly on a TurboVap (Zymark) to remove the organic solvent. The sample extracts were stored overnight under nitrogen before preparation for analysis.

#### Ultrahigh-performance liquid chromatography-tandem mass spectroscopy (UPLC–MS/MS)

All methods utilized a Waters ACQUITY ultra-performance liquid chromatography system and a Thermo Scientific Q-Exactive high-resolution/accurate mass spectrometer interfaced with a heated ESI source and an Orbitrap mass analyzer operated at 35,000 mass resolution. The sample extract was dried and then reconstituted in solvents compatible to each of the four methods. Each reconstitution solvent contained a series of standards at fixed concentrations to ensure injection and chromatographic consistency. One aliquot was analyzed using acidic positive ion conditions, chromatographically optimized for more hydrophilic compounds. In this method, the extract was gradient eluted from a C18 column (Waters UPLC BEH C18-2.1 × 100 mm, 1.7 μm) using water and methanol, containing 0.05% perfluoropentanoic acid and 0.1% formic acid. Another aliquot was also analyzed using acidic positive ion conditions; however, it was chromatographically optimized for more hydrophobic compounds. In this method, the extract was gradient eluted from the same aforementioned C18 column using methanol, acetonitrile, water, 0.05% perfluoropentanoic acid, and 0.01% formic acid and was operated at an overall higher organic content. Another aliquot was analyzed using basic negative ion optimized conditions using a separate dedicated C18 column. The basic extracts were gradient eluted from the column using methanol and water, amended with 6.5 mM ammonium bicarbonate at pH 8. The fourth aliquot was analyzed via negative ionization following elution from a HILIC column (Waters UPLC BEH Amide 2.1 × 150 mm, 1.7 μm) using a gradient consisting of water and acetonitrile with 10 mM ammonium formate, pH 10.8. Raw data was extracted, peak-identified, and QC processed using Metabolon’s hardware and software. Compounds were identified by comparison to library entries of purified standards or recurrent unknown entities based on retention time/index, mass to charge ratio (m/z), and chromatographic data, and peaks were quantified using area-under-the-curve.

#### Statistical analysis

The dataset comprises a total of 774 biochemicals. Metabolic profiles were quantified in terms of relative abundance and median scaled to 1. Following log transformation and imputation of missing values, if any, with the minimum observed value for each compound, two-way ANOVA contrast were used to identify biochemicals that differed significantly between experimental groups. An FDR adjusted*-P* value (i.e., *q*-value) is calculated to take into account the multiple comparisons that normally occur in metabolomic-based studies, and all metabolites with *q*-value < 0.05 were included.

### RNA-seq analysis

Mouse liver tissue samples were submitted to the University of Wisconsin Biotechnology Center (UWBC) Gene Expression Center for total RNA extraction. In a 96-well format, tissue samples were lysed using QIAzol Lysis Reagent (Qiagen, Hilden, Germany) and the TissueLyser. Following phase separation by centrifugation, the aqueous phase was recovered, ethanol was added, and the solution was added to an RNeasy 96 Universal Tissue plate. Plate was processed following the RNeasy 96 Universal Tissue protocol. An on-column DNase treatment step was included. RNA was eluted in nuclease-free water. Each sample was quantified and analyzed on a NanoDrop One Spectrophotometer (Thermo Fisher Scientific, Waltham, MA, USA) and Agilent 2100 Bioanalzyer (Santa Clara, CA, USA) for purity and integrity, respectively.

Total RNA samples that met the Illumina sample input guidelines were prepared according the TruSeq® Stranded mRNA Sample Preparation Guide (Rev. E) using the Illumina® TruSeq® Stranded mRNA Sample Preparation kit (Illumina Inc., San Diego, CA, USA). For each library preparation, mRNA was purified from 1000 ng total RNA using poly-T oligo-attached magnetic beads. Subsequently, each poly-A-enriched sample was fragmented using divalent cations under elevated temperature. The mRNA fragments were converted to double-stranded cDNA (ds cDNA) using SuperScript II (Invitrogen, Carlsbad, CA, USA), RNaseH, and DNA Pol I, primed by random primers. The ds cDNA was purified with AMPure XP beads (Agencourt, Beckman Coulter). The cDNA products were incubated with Klenow DNA Polymerase to add an “A” base (Adenine) to the 3′ end of the blunt DNA fragments. DNA fragments were ligated to unique dual index (UDI) adapters (IDT for Illumina- TruSeq RNA UD Index- catalog 20022371, IDT for Illumina - Nextera DNA Unique Dual Indexes, Set A and custom synthesized UDIs), which have a single “T” base (Thymine) overhang at their 3′ end. The adapter-ligated DNA products were purified with AMPure XP beads. Adapter-ligated DNA was amplified in a Linker Mediated PCR reaction (LM-PCR) for 10 cycles using Phusion TM DNA Polymerase and Illumina’s PE genomic DNA primer set followed by purification with AMPure XP beads. Finally, the quality and quantity of the finished libraries were assessed using an Agilent DNA1000 chip (Agilent Technologies, Inc., Santa Clara, CA, USA) and Qubit® dsDNA HS Assay Kit (Invitrogen, Carlsbad, CA, USA), respectively. Libraries were standardized to 2 nM. Paired-end 2 × 150 bp sequencing was performed, using standard SBS chemistry on an Illumina NovaSeq6000 sequencer. Images were analyzed using the standard Illumina Pipeline, version 1.8.2.

High-quality reads were obtained after removal of adaptor sequences and high content of unknown bases. Filtered reads were mapped to mouse genome reference GRCm38.p6 from Ensemble (release 97) database using Spliced Transcripts Alignment to Reference tool (STAR, v2.7.2a). Further quantification of mapped transcript reads to the reference was performed using featureCounts tool (v1.6.4). Readcount data was analyzed for differential gene expression using edgeR Bioconductor package (version 3.28.0) performing the generalized linear model quasi-likelihood F-test from empirical Bayes methods to estimate gene-specific biological variation [96]. Quasi-likelihood method accounts for uncertainty in dispersion estimation, therefore, gives stricter error rate control which makes it ideal for differential expression analyses of our RNA-seq data [96]. Filtering of low expressed genes was performed by keeping counts in a minimum number of samples computed through the built-in function “filterByExpr”. Normalization for RNA composition effect was performed in order to compare relative changes in expression levels between conditions using trimmed mean of *M*-values (TMM) between each pair of samples [97]. Biological and technical variability estimation was performed by analyzing sample replicates. Negative binomial generalized linear models were fitted and tagwise dispersion estimates were obtained using Cox-Reid profile-adjusted likelihood method in order to determine differential expression. Differentially expressed genes (DEGs) were obtained after filtering by logarithmic fold-change 1 and − 1, *P* value 0.05, and false discovery rate (FDR adjusted*-P*) 0.05, yielding 55,573 genes in total. Gene Ontology (GO) functional enrichment analysis for biological process (BP), cellular component (CC), molecular function (MF), and Kyoto Encyclopedia of Genes and Genomes (KEGG) pathways was obtained as part of the downstream procedure to interpret the differential expression of genes. We performed Gene Ontology and KEGG pathways enrichment analyses for the differentially expressed genes. The resulting gene representation data was then filtered to include only GO terms or KEGG pathways with at least 10 annotated genes; a minimum of 5 genes that were significantly regulated and a P value (≥ 5).

### Mass spectrometry analysis of post-translational modification (PTM) of histones from liver

#### Tissue fractionation and histone extraction and label-free chemical derivatization from liver

Tissue fractionation and histone acid extraction was performed using previously published protocols [72, 98]. Briefly, 50–100 mg of frozen mice liver tissue (*n* = 32, 4 samples per treatment) was dounce-homogenized on ice in a hypotonic lysis buffer containing histone deacetylase and protease inhibitors, followed by the centrifugation to pellet nuclei. Histones were acid extracted, and a Bradford assay was performed to quantify protein yield. In total, 5 μg of dried histone extract was then subjected to hybrid chemical derivatization [99] using heavy acetic anhydride. This procedure was followed by trypsinization for 4 h and derivatization of newly generated peptide N-termini with phenylisocyanate (PIC). Finally, labelled histones were desalted using C18 stage tips.

#### Nano-liquid chromatography and electrospray ionization tandem MS

For each sample, derivatized histone peptides were injected onto a Dionex Ultimate3000 nanoflow HPLC with a Waters nanoAcquity UPLC C18 column (100 m × 150 mm, 3 m) coupled to a Thermo Fisher Q-Exactive mass spectrometer at 700 nL/min. Mobile phase consisted of water + 0.1% formic acid (A) and acetonitrile + 0.1% formic acid (B). Histone peptides were resolved with a 2-step linear gradient of 2 to 25% mobile phase B over 60 min followed by 25 to 40% mobile phase B over 15 min. Data was acquired using data-independent acquisition (DIA) mode. The mass spectrometer was operated with a MS1 scan at resolution = 35,000, automatic gain control target = 1 × 106, and scan range = 390–910 m/z, followed by a DIA scan with a loop count of 10. DIA settings were as follows: window size = 10 m/z, resolution = 17,500, automatic gain control target = 1 × 106, DIA maximum fill time = AUTO, and normalized collision energy = 30. For each cycle, one full MS1 scan was followed by 10 MS2 scans using an isolation window size of 10 m/z.

#### Histone PTM quantification

EpiProfile 2.0 was used for quantification of histone PTMs [100]. The R script provided by Denu lab (https://github.com/DenuLab/HistoneAnalysisWorkflow) was used to perform data cleaning, normalization, statistical analysis, and visualization.

### Data processing for microbiome, metabolome, and transcriptome

#### Normalization/transformation/filtering

For the taxa that were unclassified at the genus level, their identities at higher levels were used. We combined all ASVs belonging to the same genus and filtered out the genera that appear in fewer than 20% of total samples, leaving 5 phyla, 11 classes, 11 orders, 25 families, and 45 genera. For the metabolome data, we normalized biochemicals by using inverse normal transformation and transformed variables that did not follow a normal distribution (Shapiro-Wilk test *P* < 0.05) were removed, resulting in 712 biochemicals. The RNA-seq data were normalized with voom methodology [101]. The voom method estimates the mean-variance relationship and computes appropriate observation-level weights to transform count data to log2-counts per million. We used median absolute deviation to measure the variability of each gene because two genes without notable variance between samples will be highly correlated. As a heuristic cut-off, the top 5000 most variant genes had been used in the downstream analysis.

#### Weighted correlation network analysis (WGCNA) for metabolome and transcriptome

In order to group the biochemicals that were highly correlated, we built the co-expression network using WGCNA [102]. The WGCNA is an efficient and robust method in grouping metabolomic and transcriptomic data [103, 104] and allowed us to summarize each module by its module eigenvalue. A one-sided Fisher test was used to determine if a pathway was enriched within the turquoise and blue modules in metabolomic data. *P* values were then adjusted using Benjamini-Hochberg method, and a cut-off of *P* < 0.05 and FDR adjusted*-P* < 0.05 were chosen to determine if a pathway was significantly enriched. We used Pearson’s correlation between expression profile of each gene and module eigenvalue to identify module membership. Using the module eigenvalue, the module-traits relationships were estimated by calculating Pearson’s correlations between the module eigenvalue and the traits of interest. We considered 0.90 as a correlation cut-off to choose soft-thresholding power and set the minimal module size as 20. For metabolome, the metabolites were clustered into 8 modules plus 43 unclustered metabolites. The transformed values of the unclustered metabolites were combined with standardized module eigenvalues in the following analysis. For the transcriptome data, 14 modules (defined as clusters of highly interconnected genes) were identified by using DynamicTree Cut algorithm. WGCNA led to 14 different modules by using DynamicTree Cut algorithm. Over-representation of genes in the blue module was characterized based on gene ontology biological process and KEGG pathways using clusterProfiler [105].

### Microbiome association with host -omics

We applied the sparse linear log-contrast model [106] to pinpoint important genera that are associated with individual metabolite/gene. In this model, the host omics variable is the response and the genus-level microbial taxa are compositional covariates. The sparse linear log-contrast model respects the compositional nature of the microbiome data and avoids choosing an arbitrary reference taxon, in which the unit-sum constraint on the compositional vector is translated into the zero-sum constraint on the association coefficients across taxa in log ratio scale. In our analysis, we used 10-fold cross validation to choose the tuning parameter. To obtain stable selection results, we generated 100 bootstrap samples and used the same cross validation procedure to select the genera. We also followed the stability selection approach [107] to assess the stability of the selected genera, where 100 subsamples of half sample size were taken to compute the selection probabilities. In the association network (Figs. 6 and 9), we kept genera with stability selection probability larger than 0.85 and filter out the lowly associated genera which the absolute value of coefficient is less than 0.1.

### Causal mediation analysis

We performed mediation analyses to investigate how the post-intervention gut microbiome/metabolites/gene expressions may mediate the effect of fecal colonization on various phenotypes (adiposity, liver TG, glucose) under a given diet. We employed the causal mediation model with batch effects as confounder, SubA/B microbial communities as exposure, and different phenotypes as outcome. All the mediation effect hypotheses were tested by using the resampling method. Below we described two approaches for testing microbiome mediation effects on the global community level and on the subcompositions defined on the taxonomy tree. For the metabolites and genes, we applied mediation analysis to the corresponding WGCNA modules.

#### Beta-diversity mediation analysis for microbiome

For beta-diversity distance matrices, we performed the distance-based mediation test by using the MedTest package in R language [108]. The Jaccard and unweighted UniFrac distance matrices were calculated based on the rarefied genus-level abundance matrix (rarefied to the minimum sequence depth) to reduce potential sequence depth-dependent bias.

#### Tree-based mediation analysis microbiome

We used maximum round error 0.5 to replace 0 [109] in full-composition abundance matrix, then calculated the sub-composition relative abundance matrix for each high-rank internal node. We removed extremely rare taxa (only detected in 10% of the observations or less). We selected the most abundant taxa in the relative abundance matrix as the reference taxa and took additive logarithm transformation on the compositional data so that the transformed data could be considered as multivariate variables. Finally, we applied the causal mediation model on each high-rank internal node in the taxonomy tree.

#### Metabolite module/gene module mediation analysis

In order to identify the microbial community status effect on different phenotypes which were transmitted through the metabolite modules/gene modules, we applied causal mediation model by considering the corresponding WGCNA modules as multiple independent mediators.

## Supplementary Information

**Additional file 1: Fig. S1.** Screening phase. 16S rRNA gene sequence analysis of fecal samples from human donors and recipient mice. A. Principal Coordinates Analysis (PCoA) of unweighted UniFrac (uwUF) distances from eight human fecal samples collected for Wisconsin Longevity Study. B. Bacterial relative abundance summarized at the phylum level. C. Percentage of genera shared between each donor fecal sample and its corresponding recipient mice cecal samples. Number of mice colonized for each fecal donor is reported under each bar. D. Percent relative abundance of the fecal donor community captured in the mouse cecal samples. E. UwUF distances between donor fecal samples and each engrafted cecal community. Averages of distances between corresponding human donor-mouse engrafted community are indicated as DONOR. Average of uwUF distances between non-matched donor-mouse community are indicated as OTHER. F. PCoA of uwUF distances of the eight human fecal samples engrafted in the mouse cecum. Circles with the same colors indicate biological replicates colonized with the same community. G. Alpha-diversity as determined by Faith’s phylogenetic diversity of each of the eight engrafted communities. ***P < 0.001.

**Additional file 2: Fig. S2.** Variation in cecal short-chain fatty acids among transplanted communities. Cecal levels of (A) butyrate; (B) acetate; and (C) propionate (μmoles/g wet weight) for each the eight transplanted groups of mice described in Fig. S1.

**Additional file 3: Fig. S3.** Variation in predicted metabolic capacity among engrafted gut communities. Principal Coordinates Analysis (PCoA) of Bray Curtis dissimilarity using the PICRUSt2 predicted metabolic functions of the eight transplanted human microbiota samples used in this study. Circles with the same colors indicate biological replicates colonized with the same community.

**Additional file 4: Fig. S4.** Characterization of transplanted communities in mice. 16S rRNA gene sequence analysis of engrafted cecal communities. Germ-free mice were colonized with SubA or SubB and exposed to one of four diets containing a different type of fiber; (i) Cellulose; (ii) Inulin; (iii) Pectin; or (iv) Assorted fiber. A. Heatmap showing presence/absence of bacterial taxa in the gut of transplanted animals across the four different diets. Red indicates presence and black absence. Each column represents an individual mouse. B. Alpha diversity (Shannon Index) of SubA and SubB communities after dietary intervention. *P < 0.05, **P < 0.01, ***P < 0.001 ****P < 0.0001.

**Additional file 5: Fig. S5.** Differences in gut microbiota between SubA- and SubB-colonized animals across the different diets used. Weighted UniFrac distances between fecal microbiomes of SubA and SubB colonized mice. The UniFrac matrix was permuted 999 times; *n* = 7-10 animals/microbiome/diet.

**Additional file 6: Fig. S6.** Individual effect of dietary fibers on Firmicutes to Bacteroidetes ratio. Comparison of Firmicutes and Bacteroidetes between engrafted SubA and SubB communities across different diets. A. Relative abundance of Bacteroidetes (white) and Firmicutes (black) in SubA (magenta) and SubB (yellow) colonized mice for each dietary fiber intervention. B. Firmicutes:Bacteroidetes ratio in SubA (magenta) and SubB (yellow) for the four dietary interventions. *P < 0.05, **P < 0.01, ***P < 0.001, ****P < 0.0001.

**Additional file 7: Fig. S7.** Linear discriminant analysis Effect Size (LEfSe) summary. List of taxa differentially abundant between gut community SubA (magenta) and SubB (yellow) in the four diets. LDA score (log 10) is indicated at the bottom of each graph.

**Additional file 8: Fig. S8.** Relative abundance of gut bacterial taxa for SubA and SubB. Box plots indicating relative abundance of taxa of interest relevant to the diversity, association, and mediation analyses. This figure shows relative abundance of taxa that has at least one significant difference between SubA and SubB within a dietary intervention. SubA is represented with the color magenta and SubB with the color yellow. *P < 0.05, **P < 0.01, ***P < 0.001, ****P < 0.0001.

**Additional file 9: Fig. S9.** Short Chain Fatty Acids (SCFA). Cecal levels of (A) acetate; (B) butyrate, (C) propionate and total SCFA (umoles/g wet wt) E. valerate and Branched-chain Fatty Acids (BCFA) Isobutyrate and Isovalerate of SubA (magenta) and SubB (yellow) by diet. Wilcoxon test comparison *P < 0.05, **P < 0.01, ***P < 0.001, ****P < 0.0001.

**Additional file 10: Fig. S10.** Dendrogram of serum metabolites from transplanted mice. Clustering dendrograms of 712 serum metabolites with dissimilarity based on topological overlap, together with assigned module colors. There are 9 modules that cluster different numbers of metabolites.

**Additional file 11: Fig. S11.** Correlations between Short Chain Fatty Acids and blood metabolites. Correlation matrix between metabolites consensus modules and cecal SCFAs from mice described in Fig. 1. Modules were determined based on patterns of co-abundance of metabolites using weighted correlation network analysis (WGCNA). Each of the modules was labelled with a unique color as an identifier. Each module was tested for correlation with each cecal SCFA quantified. Within each cell, upper values are correlation coefficients between module and the phenotypes; lower values are the corresponding FDR adjusted*-P* values. B. Pathways enriched in the yellow, blue and turquoise modules as determined by Fisher’s test.

**Additional file 12: Fig. S12.** Branched-Chain Fatty Acids and taxa correlation. The heatmap shows all correlations (*P* < 0.05) using Spearman method between BCFA and taxa in each dietary intervention.

**Additional file 13: Fig. S13.** Gut community-mediated epigenetic changes in liver are sensitive to dietary fiber. Abundance of histone Post-Translational Modifications (PTMs) on H3 lysines (K9, K14, K27, and K36). A. H3K9K14 peptide. B. H3K27K36 peptide (*n* = 4/community/diet). *P < 0.05, **P < 0.01; ac, acetylated; unmod, unmodified; meth1,2,3, mono- di- and try- methylated respectively; pr, propionylated.

**Additional file 14: Fig. S14.** Effect of gut community on liver histone post-translational modifications (PTMs). Heatmap show relative difference in abundance for each histone PTM quantified in liver for mice colonized with the two communities (Log2 fold-change of SubB/SubA) in each diet. *P < 0.1, **P < 0.01, ***P < 0.001 (*n* = 3-4/condition). ac, acetylated; unmod, unmodified; meth1,2,3, mono- di- and try- methylated respectively; pr, propionylated.

**Additional file 15: Fig. S15.** Dendrogram of liver transcripts from transplanted mice. Clustering dendrograms of genes with dissimilarity based on topological overlap, together with assigned module colors. There are 14 modules that cluster different numbers of transcripts.

**Additional file 16: Fig. S16.** Gene Ontology and KEGG pathway enrichment of transcripts in the blue module associated with metabolic phenotypes. A. Biological Process GO and KEGG enrichment of blue module associated with adiposity, B. Association with liver triglycerides. C. Association with glucose. Gene counts and FDR adjusted*-P* values are indicated for each enrichment box.

**Additional file 17: Table S1.** Diets used in the study.

**Additional file 18: Table S2.** Statistical analysis of metabolic phenotypes in transplanted mice.

**Additional file 19: Table S3.** Relative abundance of bacterial ASVs detected in mice. 16S rRNA gene sequence analysis of cecal samples from gnotobiotic mice colonized with SubA or SubB communities consuming one of four diets containing different types of fiber; (i) Cellulose; (ii) Inulin; (iii) Pectin; or (iv) Assorted fiber for four weeks.

**Additional file 20: Table S4.** Relative abundance of bacterial genera detected in mice. 16S rRNA gene sequence analysis of cecal samples from gnotobiotic mice colonized with SubA or SubB communities consuming one of four diets containing different type of fiber; (i) Cellulose; (ii) Inulin; (iii) Pectin; or (iv) Assorted fiber for four weeks. Taxa summarized at the genus level.

**Additional file 21: Table S5.** Mediation analysis results. For each dietary fiber intervention associated phenotype a mediator type was evaluated.

**Additional file 22: Table S6.** List of serum metabolites measured in transplanted mice. Fold-change (FC) between SubB and SubA is indicated as follow: darker green indicates FC < 1 with *P* ≤ 0.05, lighter green indicates FC < 1 with 0.05 < *P* < 0.1, darker read indicates FC ≥ 1 with *P* ≤ 0.05, and lighter red indicates FC ≥ 1 with 0.05 < *P* < 0.1*.* Two-Way ANOVA main effects: Community, diet, and their interaction are indicated in blue shaded cells when significant (*P* ≤ 0.05) ANOVA effect; light blue shaded cells indicate 0.05 < *P* < 0.10.

**Additional file 23: Table S7.** Weighted Correlation Network Analysis assignment of serum metabolites into modules. Abbreviations: MS, Metabolite Significance; p.MS., *P*-value of Metabolite Significance; MM, module membership; p.MM P-value of the module membership.

**Additional file 24: Table S8.** Serum metabolites contained in the blue and turquoise modules. Metabolites (biochemicals) are organized by pathways. Fold-change between SubB and SubA is indicated for the four diets as follow: darker green indicates FC < 1 with *P* ≤ 0.05, lighter green indicates FC < 1 with 0.05 < *P* < 0.1, darker read indicates FC ≥ 1 with *P* ≤ 0.05, and lighter red indicates FC ≥ 1 with 0.05 < *P* < 0.1. The last two columns show metabolite significance (MS) which reports the association of each metabolite with each phenotype and the corresponding *P*-value (p.MS).

**Additional file 25: Table S9.** Liver gene expression analysis. List of genes differentially expressed in the liver from mice colonized with SubB vs. SubA communities. (LogFC > 1 and < -1, *P* and FDR adjusted-*P* < 0.05).

**Additional file 26: Table S10.** Analysis of differentially expressed genes in liver. Number of differentially regulated genes contained in each listed KEGG pathway and Gene Ontology Term.

**Additional file 27: Table S11.** Weighted Correlation Network Analysis assignment of liver transcripts. Abbreviations: GS, Gene Significance; p.MS., *P*-value of Gene Significance; MM, module membership; p.MM for the *P*-value of the module membership.

**Additional file 28: Table S12.** List of liver transcripts associated with bacterial taxa. bcv.prob denotes the probability of specific taxa being selected in log-contrast model based on 100 bootstrap samples; stab.prob denotes the probability of specific taxa being selected in log-contrast model based on 100 subsamples of half sample size. refitted.coef denotes the coefficient estimation in the log-contrast model based on selected taxa listed in this table.

**Additional file 29.** **Supplemental results.** Effects of microbiota-fiber interactions on liver histone posttranslational modifications.

## Data Availability

The data reported in this paper are accessible in the NCBI Short Read Archive (SRA) under accession ID PRJNA665643 and European Nucleotide Archive (ENA) accession number PRJEB40242. Original R scripts and data used for statistical analysis are available in GitHub (https://github.com/KiRinHong/fiberNmicrobiome).

## Abbreviations

ANOVA: Analysis of variance
Af: Assorted fiber
ASV: Amplicon sequence variant
BCAA: Branched-chain amino acid
BCFA: Branched-chain fatty acids
BP: Biological process
C: Cellulose
CC: Cellular component
DEGs: Differentially expressed genes
ds cDNA: Double-stranded cDNA
F/B: Firmicutes:Bacteroidetes ratio
FAST: Fecal aliquot straw technique
FC: Fold-change
FDR: False discovery rate
GF: Germ-free
GO: Gene ontology
GSH: Glutathione
I: Inulin
KEGG: Kyoto Encyclopedia of Genes and Genomes
LEfSe: Linear discriminant analysis effect size
MACs: Microbiota-accessible carbohydrates
MF: Molecular function
*ob/ob*: Genetically obese
P: Pectin
PC: Phosphatidylcholine
PCoA: Principal coordinates analysis
PD: Phylogenetic diversity
PERMANOVA: Permutational multivariate analysis of variance
PPAR: Peroxisome proliferator-activated receptor
PTMs: Post-translational modifications
RNA-seq: RNA sequencing
RS: Resistant starch
SCFAs: Short-chain fatty acids
scFOS: Short-chain fructo-oligosaccharides
SubA: Subject A
SubB: Subject B
TG: Liver triglycerides
TMM: Trimmed mean of M-values
TNF-α: Tumor necrosis factor alpha
TRP: Transient receptor potential
UPLC–MS/MS: Ultrahigh-performance liquid chromatography-tandem mass spectroscopy
uwUF: Unweighted UniFrac
WGCNA: Weighted correlation network analysis
WLS: Wisconsin Longitudinal Study
wUF: Weighted UniFrac
