# Supplementary figures and images for: Gut microbiome variation modulates the effects of dietary fiber on host metabolism
Source: Microbiome. 2021 May 20;9:117. doi: 10.1186/s40168-021-01061-6 (PMC8138933; doi:10.1186/s40168-021-01061-6)

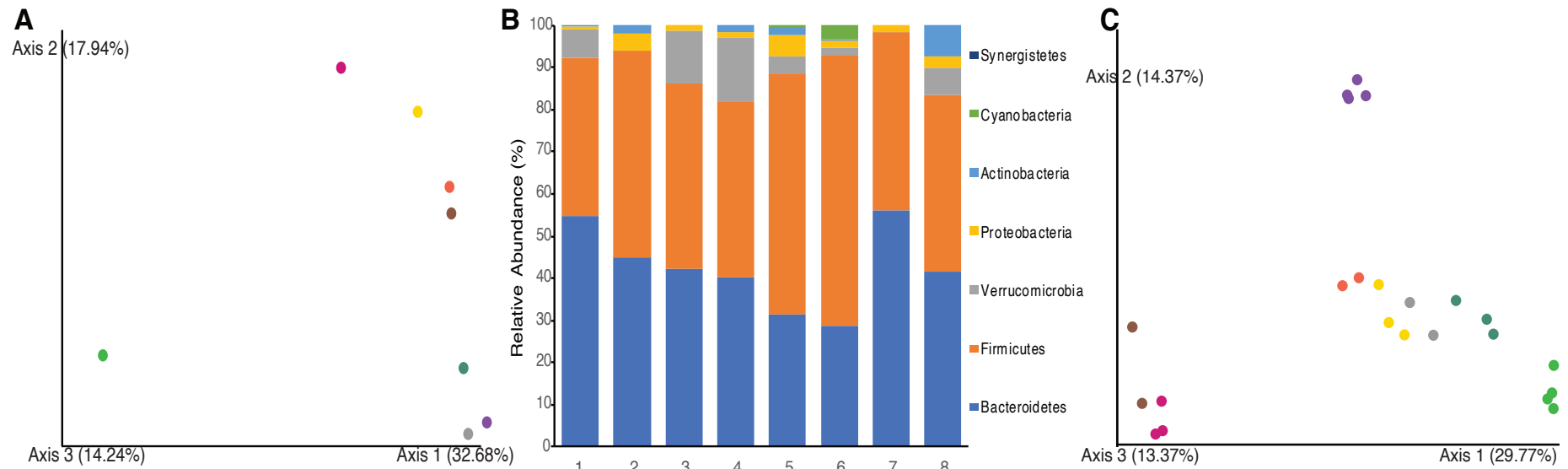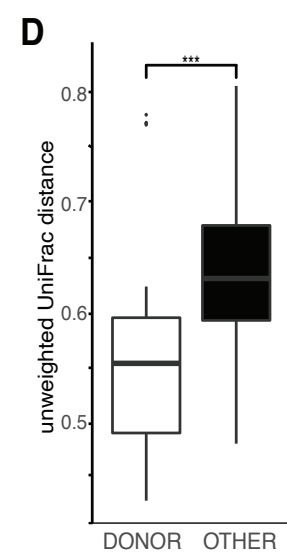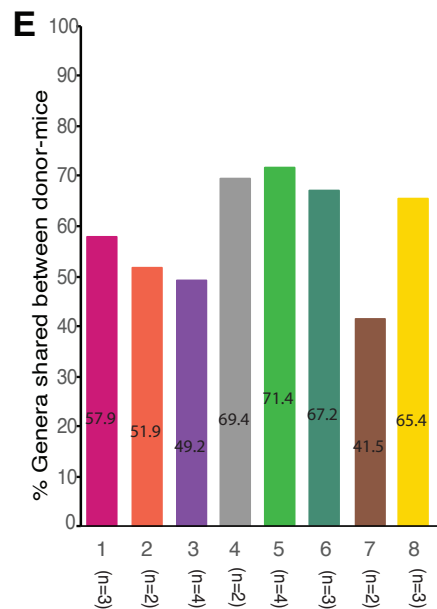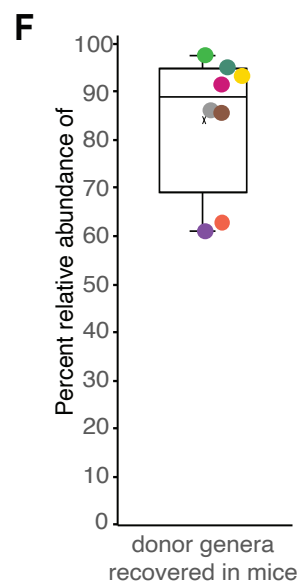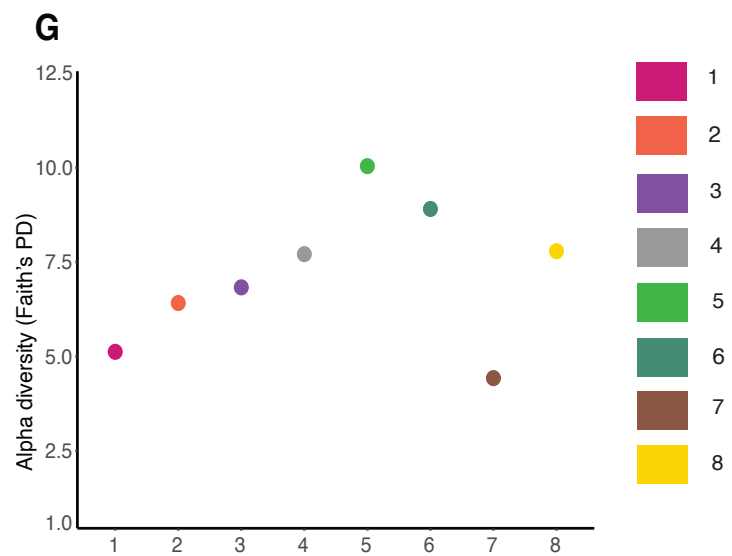

Supplement: Supplementary file 2 — Additional file 1: Fig. S1. Screening phase. 16S rRNA gene sequence analysis of fecal samples from human donors and recipient mice. A. Principal Coordinates Analysis (PCoA) of unweighted UniFrac (uwUF) distances from eight human fecal samples collected for Wisconsin Longevity Study. B. Bacterial relative abundance summarized at the phylum level. C. Percentage of genera shared between each donor fecal sample and its corresponding recipient mice cecal samples. Number of mice colonized for each fecal donor is reported under each bar. D. Percent relative abundance of the fecal donor community captured in the mouse cecal samples. E. UwUF distances between donor fecal samples and each engrafted cecal community. Averages of distances between corresponding human donor-mouse engrafted community are indicated as DONOR. Average of uwUF distances between non-matched donor-mouse community are indicated as OTHER. F. PCoA of uwUF distances of the eight human fecal samples engrafted in the mouse cecum. Circles with the same colors indicate biological replicates colonized with the same community. G. Alpha-diversity as determined by Faith’s phylogenetic diversity of each of the eight engrafted communities. ***P < 0.001. [file 40168_2021_1061_MOESM2_ESM.pdf]

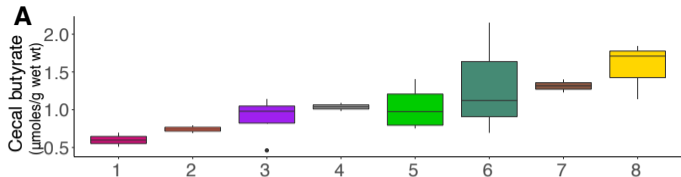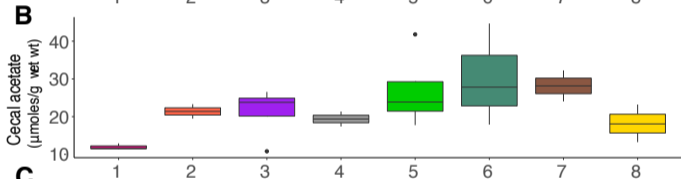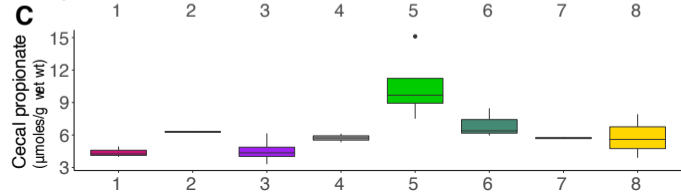

Supplement: Supplementary file 3 — Additional file 2: Fig. S2. Variation in cecal short-chain fatty acids among transplanted communities. Cecal levels of (A) butyrate; (B) acetate; and (C) propionate (μmoles/g wet weight) for each the eight transplanted groups of mice described in Fig. S1. [file 40168_2021_1061_MOESM3_ESM.pdf]

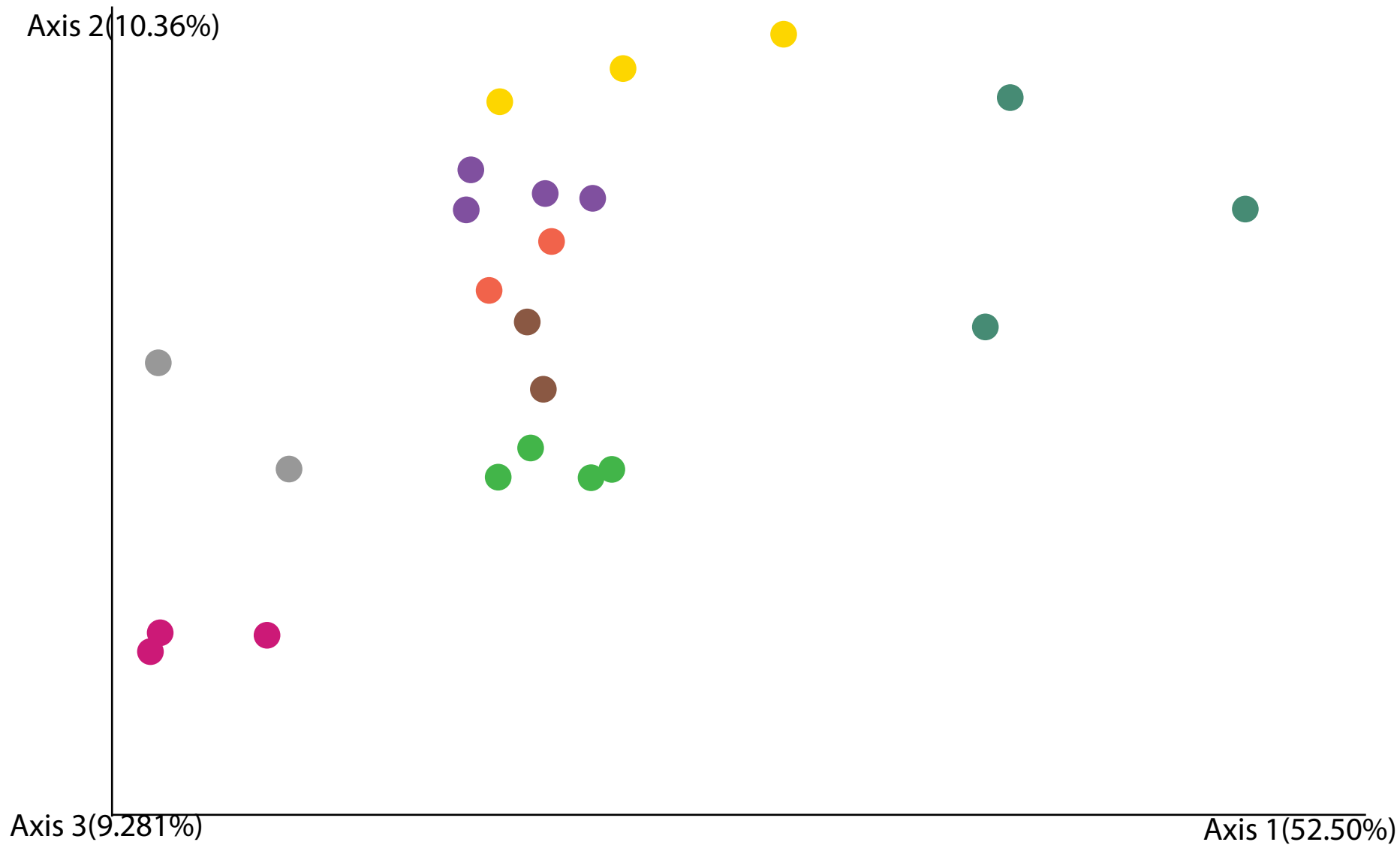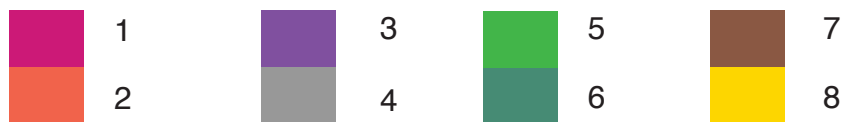

Supplement: Supplementary file 4 — Additional file 3: Fig. S3. Variation in predicted metabolic capacity among engrafted gut communities. Principal Coordinates Analysis (PCoA) of Bray Curtis dissimilarity using the PICRUSt2 predicted metabolic functions of the eight transplanted human microbiota samples used in this study. Circles with the same colors indicate biological replicates colonized with the same community. [file 40168_2021_1061_MOESM4_ESM.pdf]

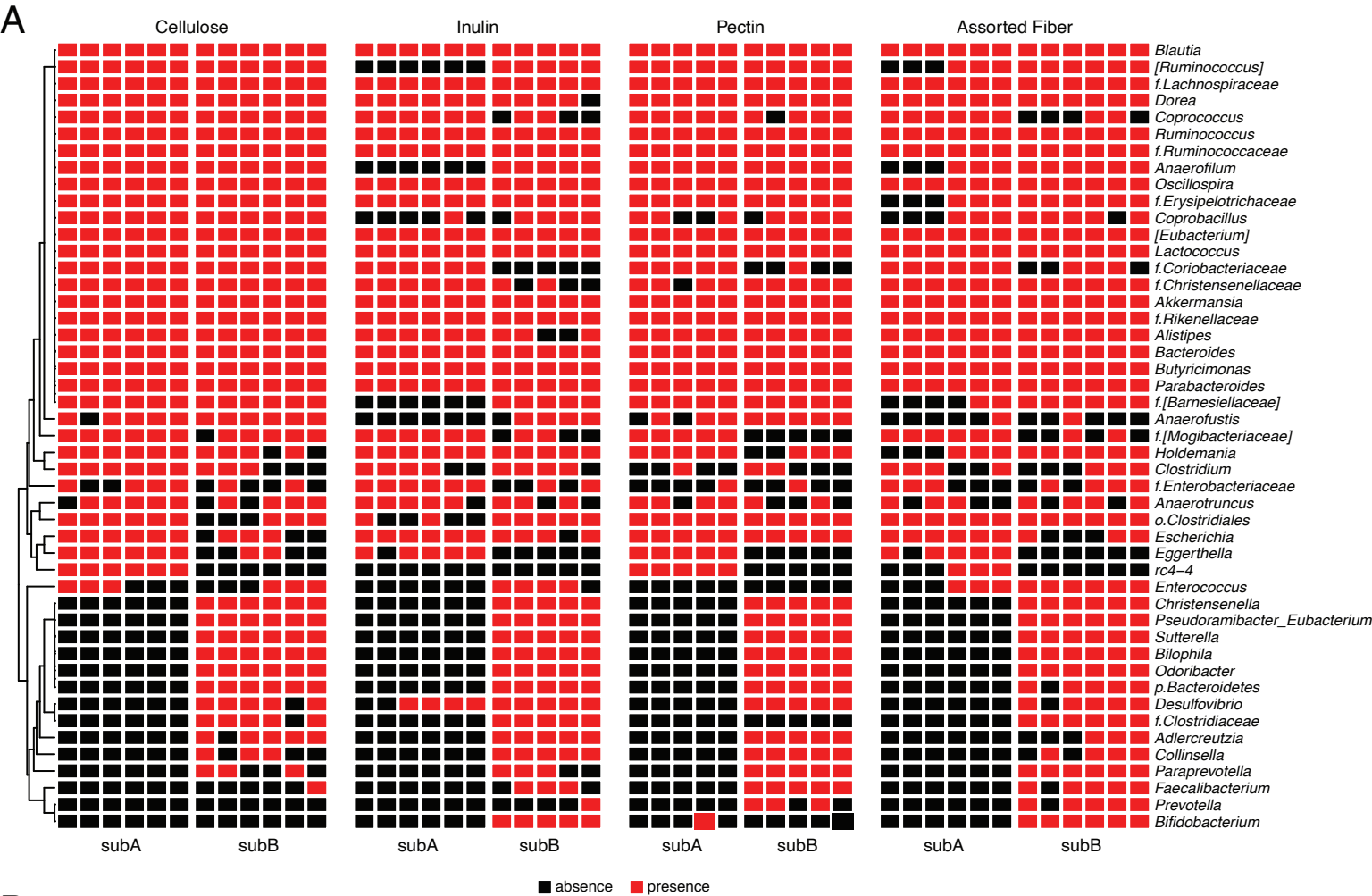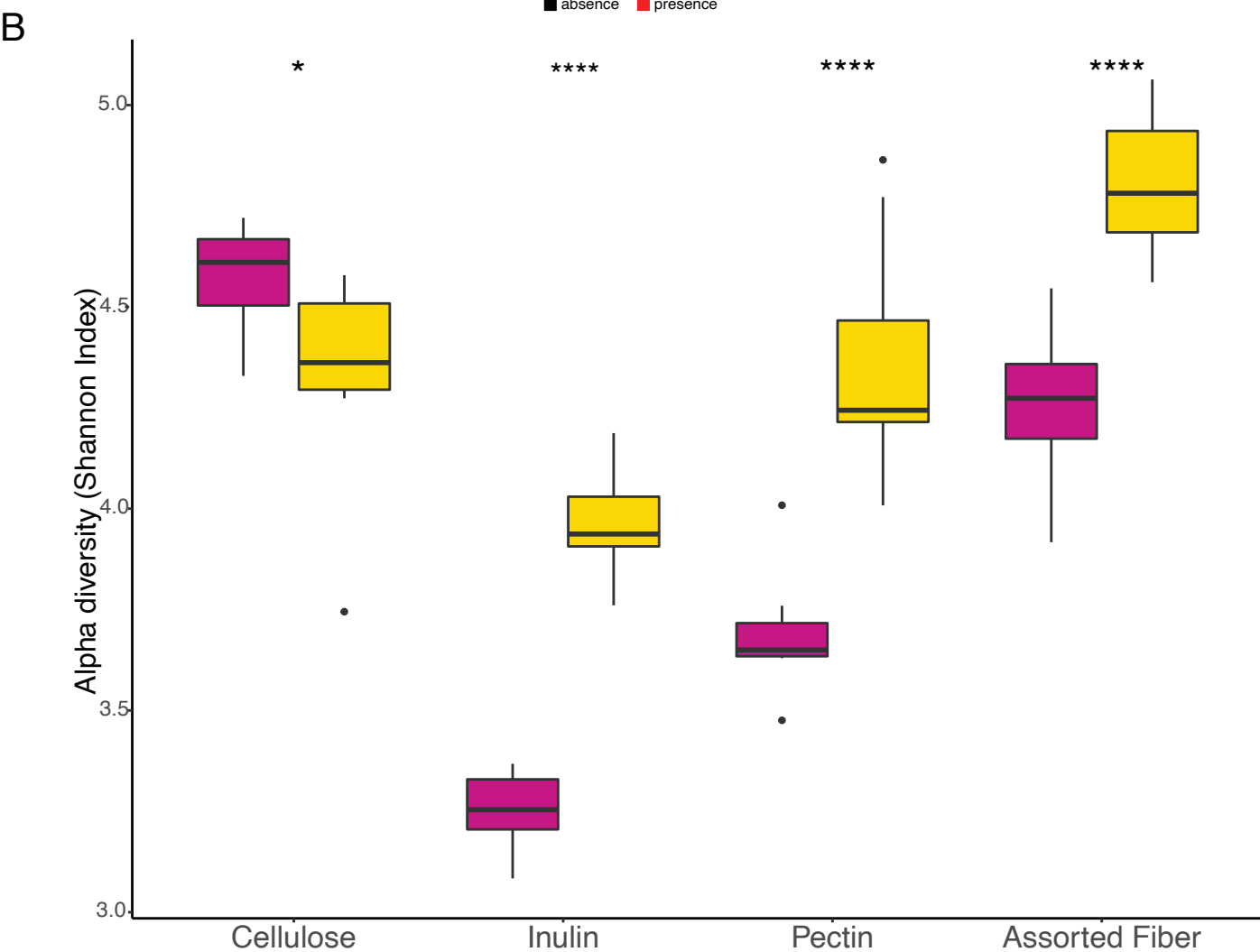

Supplement: Supplementary file 5 — Additional file 4: Fig. S4. Characterization of transplanted communities in mice. 16S rRNA gene sequence analysis of engrafted cecal communities. Germ-free mice were colonized with SubA or SubB and exposed to one of four diets containing a different type of fiber; (i) Cellulose; (ii) Inulin; (iii) Pectin; or (iv) Assorted fiber. A. Heatmap showing presence/absence of bacterial taxa in the gut of transplanted animals across the four different diets. Red indicates presence and black absence. Each column represents an individual mouse. B. Alpha diversity (Shannon Index) of SubA and SubB communities after dietary intervention. *P < 0.05, **P < 0.01, ***P < 0.001 ****P < 0.0001. [file 40168_2021_1061_MOESM5_ESM.pdf]

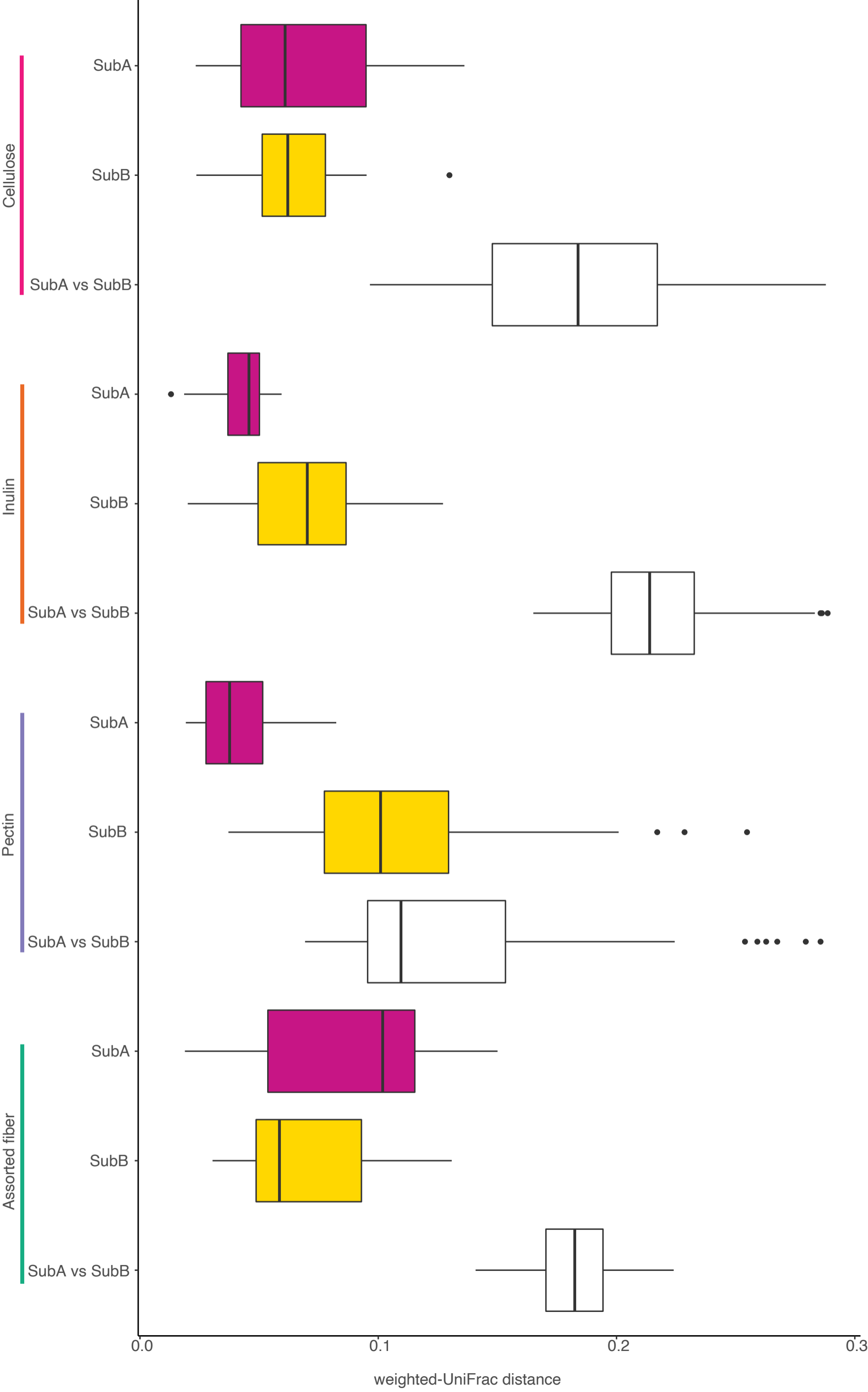

Supplement: Supplementary file 6 — Additional file 5: Fig. S5. Differences in gut microbiota between SubA- and SubB-colonized animals across the different diets used. Weighted UniFrac distances between fecal microbiomes of SubA and SubB colonized mice. The UniFrac matrix was permuted 999 times; n = 7-10 animals/microbiome/diet. [file 40168_2021_1061_MOESM6_ESM.pdf]

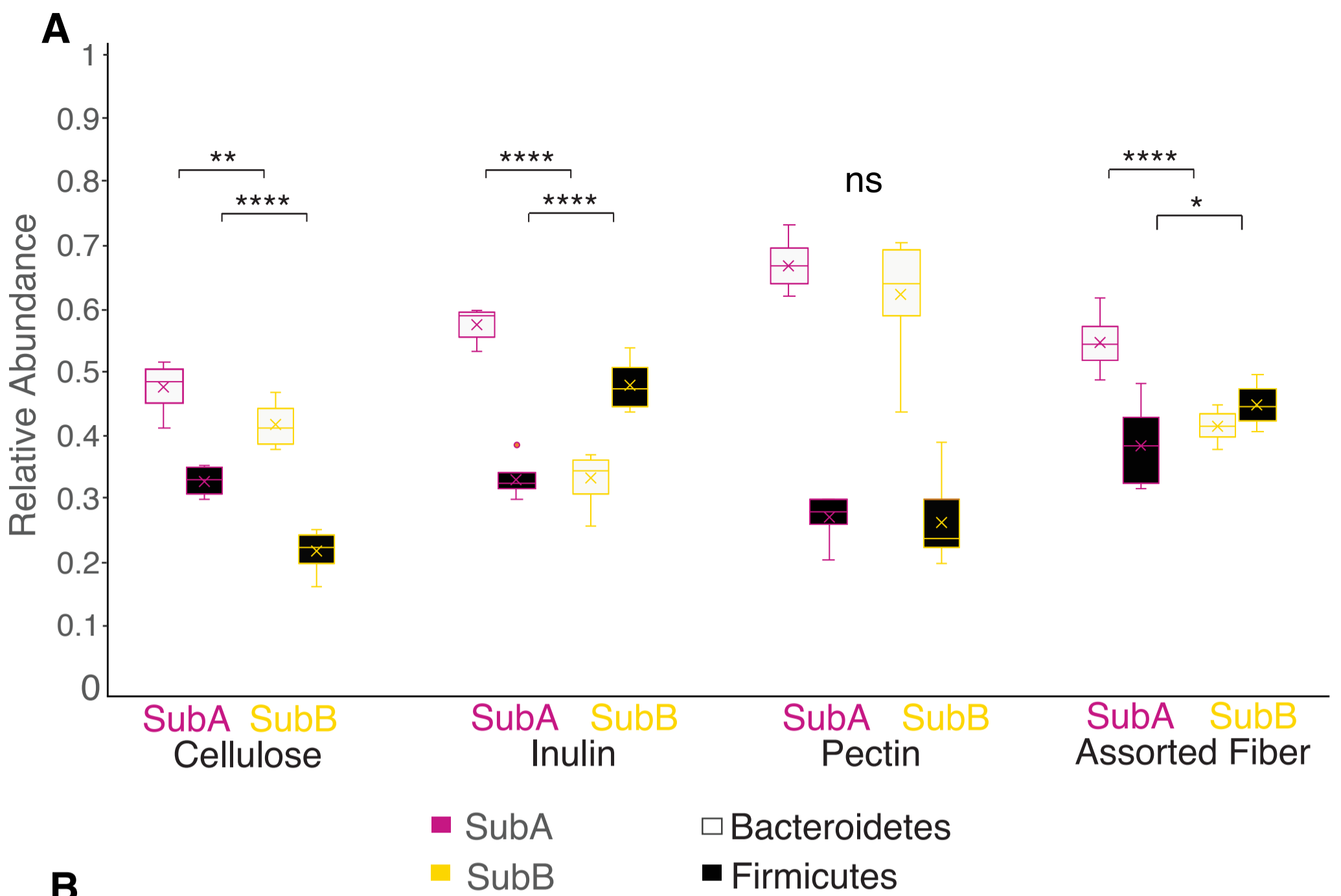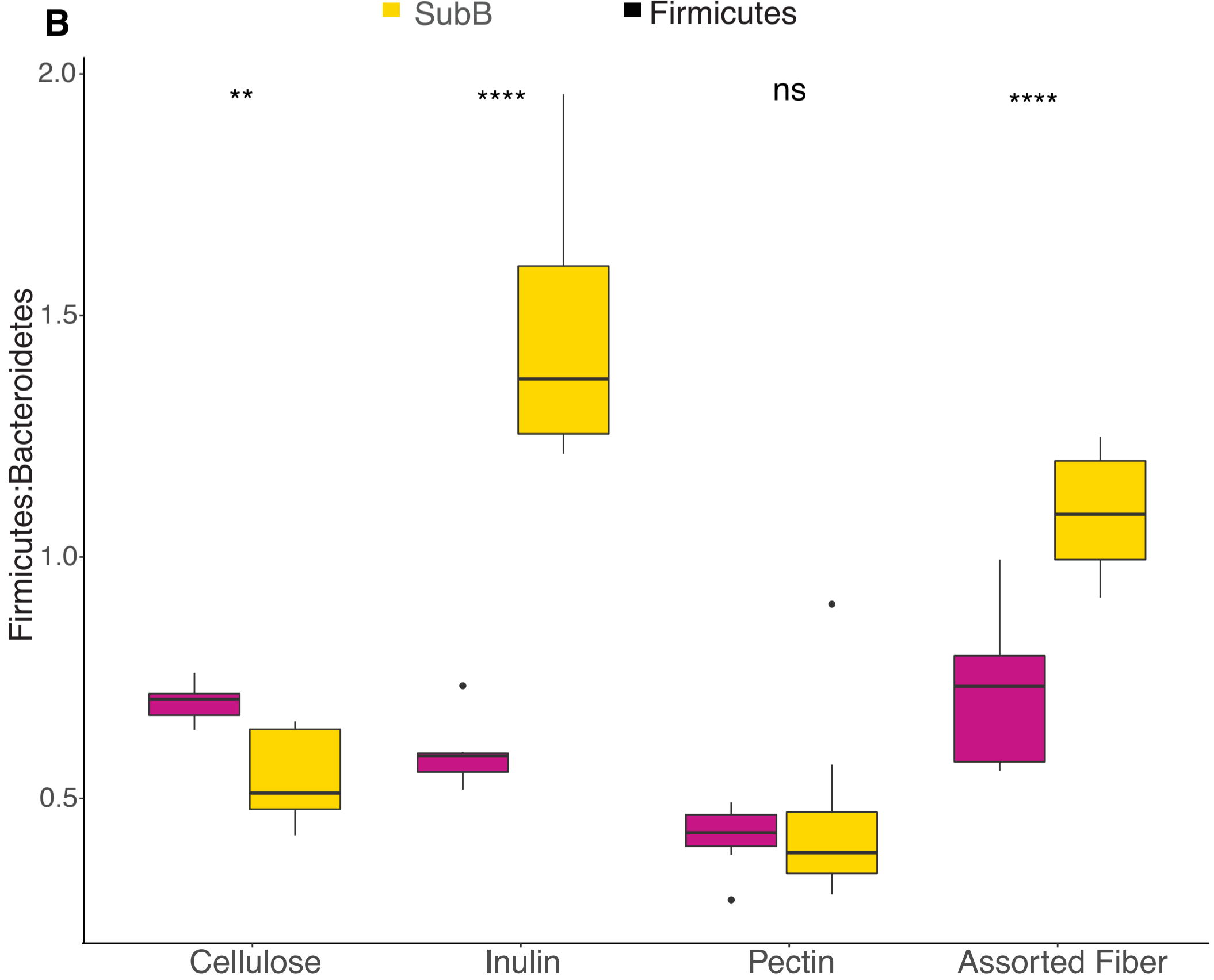

Supplement: Supplementary file 7 — Additional file 6: Fig. S6. Individual effect of dietary fibers on Firmicutes to Bacteroidetes ratio. Comparison of Firmicutes and Bacteroidetes between engrafted SubA and SubB communities across different diets. A. Relative abundance of Bacteroidetes (white) and Firmicutes (black) in SubA (magenta) and SubB (yellow) colonized mice for each dietary fiber intervention. B. Firmicutes:Bacteroidetes ratio in SubA (magenta) and SubB (yellow) for the four dietary interventions. *P < 0.05, **P < 0.01, ***P < 0.001, ****P < 0.0001. [file 40168_2021_1061_MOESM7_ESM.pdf]

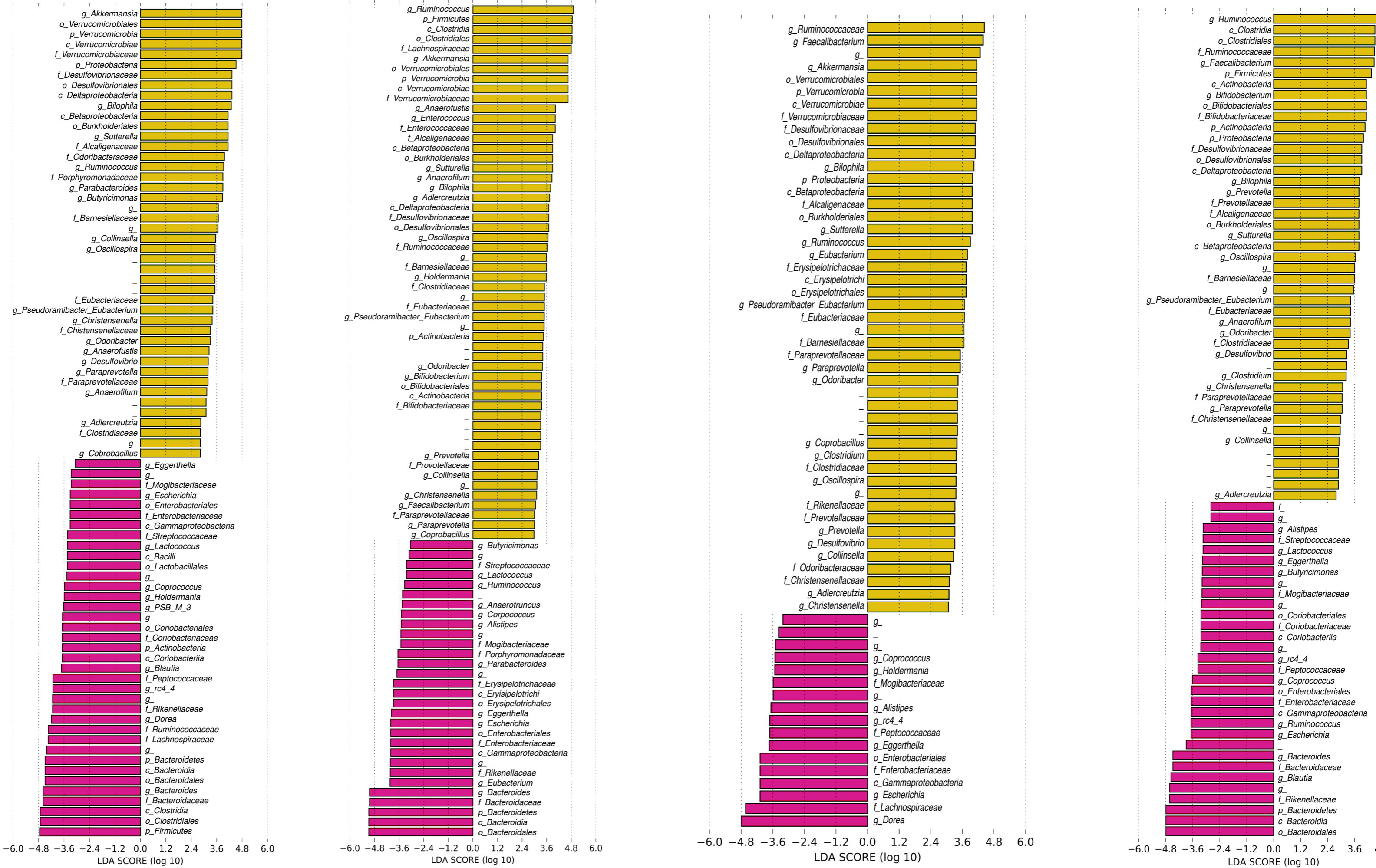

Supplement: Supplementary file 8 — Additional file 7: Fig. S7. Linear discriminant analysis Effect Size (LEfSe) summary. List of taxa differentially abundant between gut community SubA (magenta) and SubB (yellow) in the four diets. LDA score (log 10) is indicated at the bottom of each graph. [file 40168_2021_1061_MOESM8_ESM.pdf]

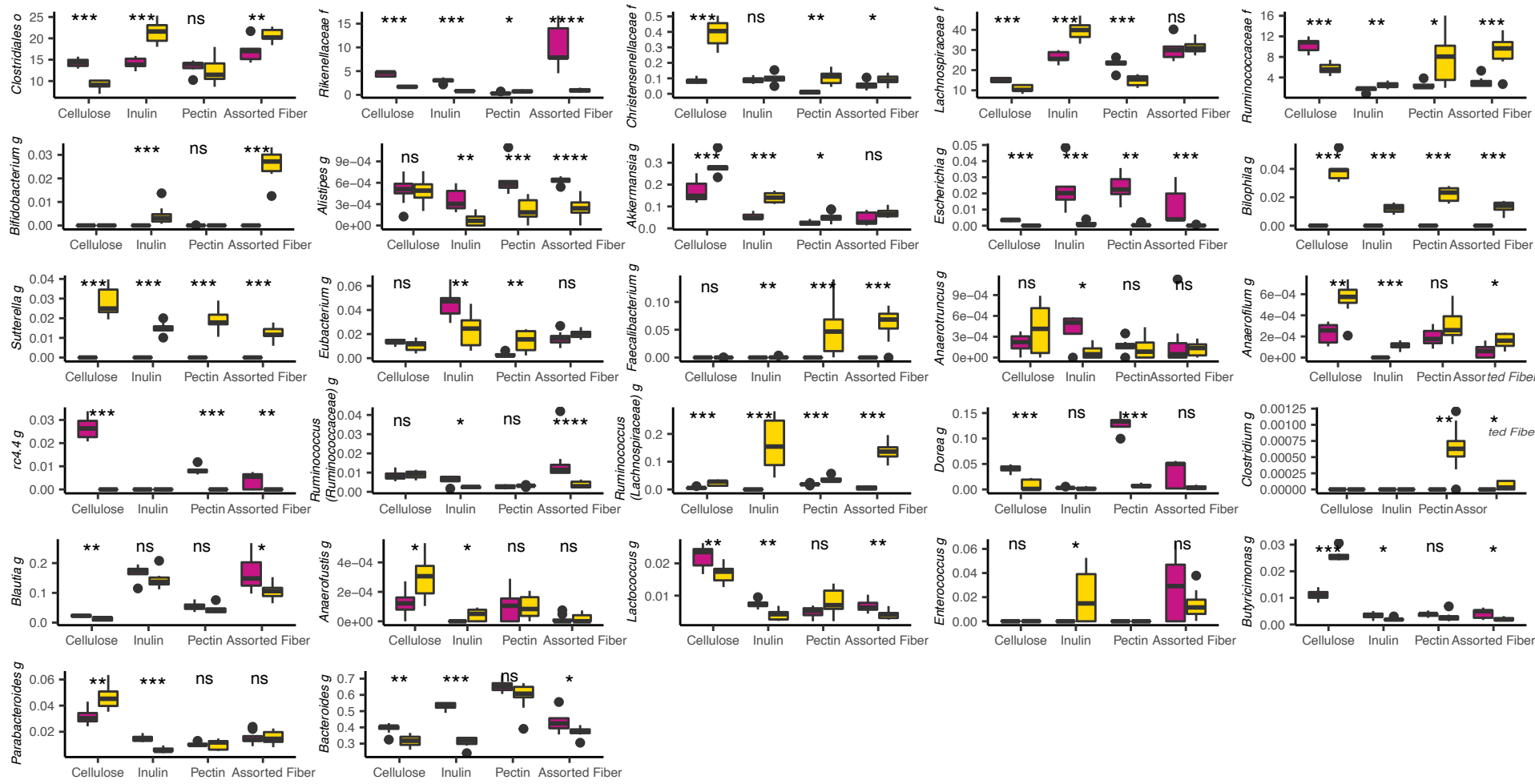

Supplement: Supplementary file 9 — Additional file 8: Fig. S8. Relative abundance of gut bacterial taxa for SubA and SubB. Box plots indicating relative abundance of taxa of interest relevant to the diversity, association, and mediation analyses. This figure shows relative abundance of taxa that has at least one significant difference between SubA and SubB within a dietary intervention. SubA is represented with the color magenta and SubB with the color yellow. *P < 0.05, **P < 0.01, ***P < 0.001, ****P < 0.0001. [file 40168_2021_1061_MOESM9_ESM.pdf]

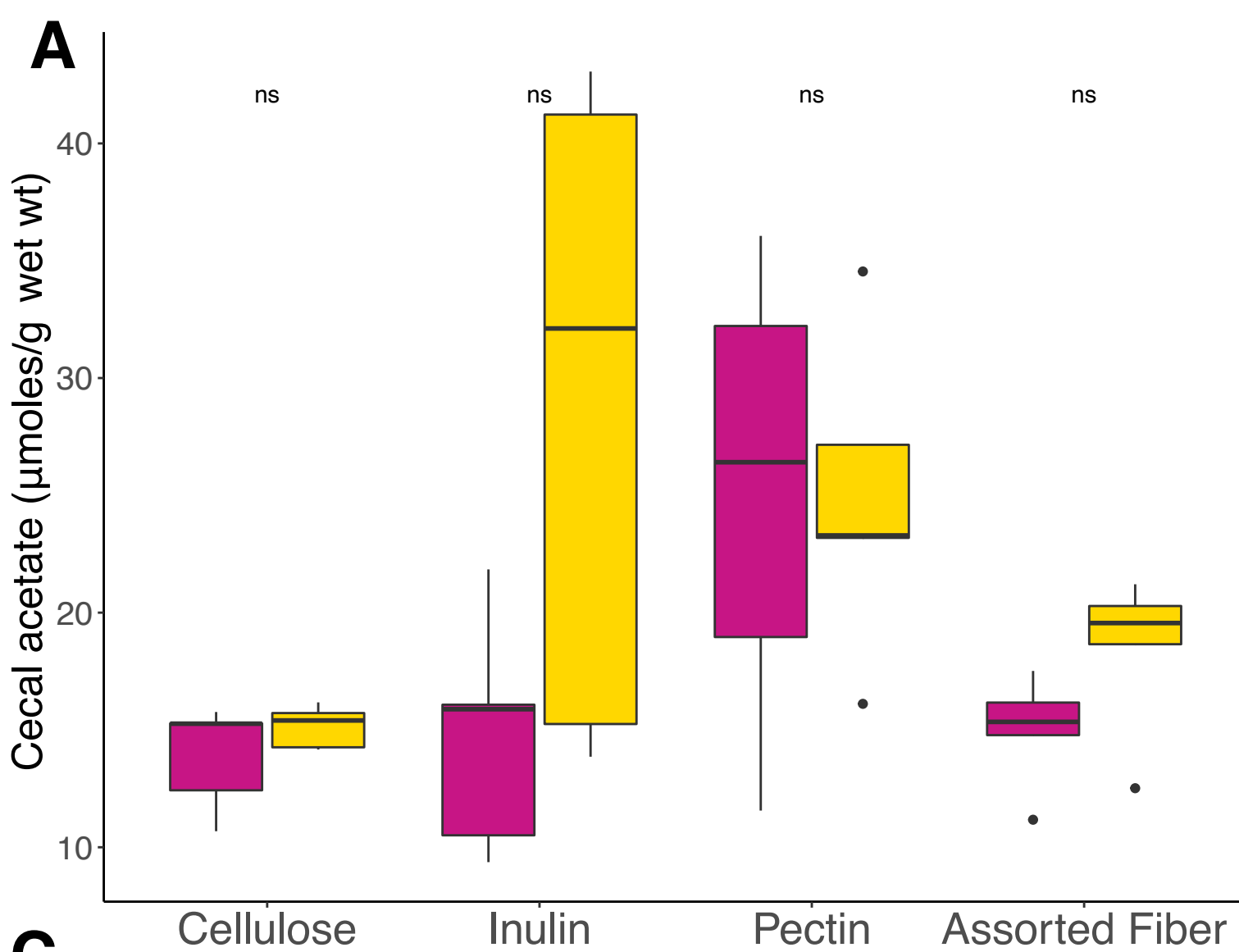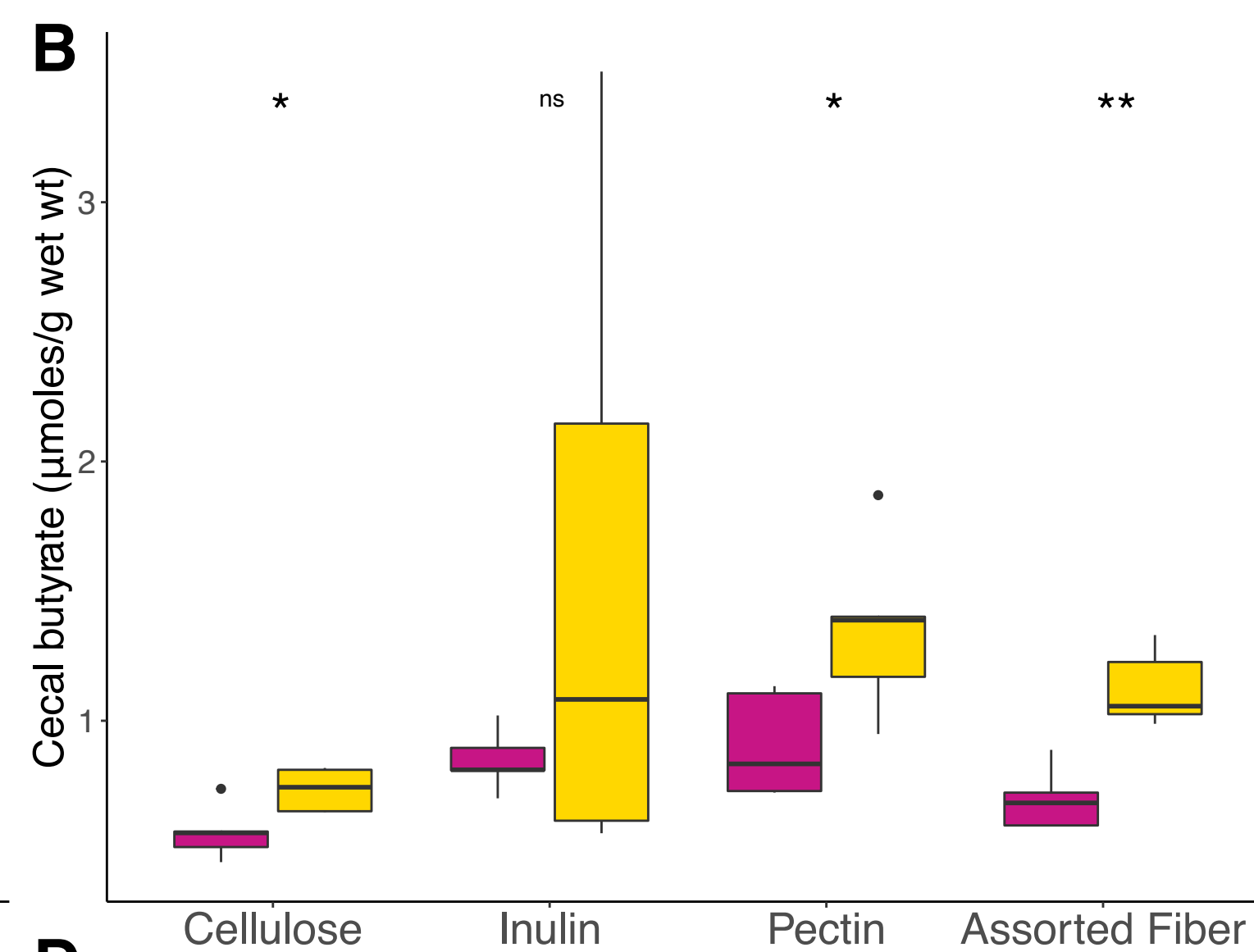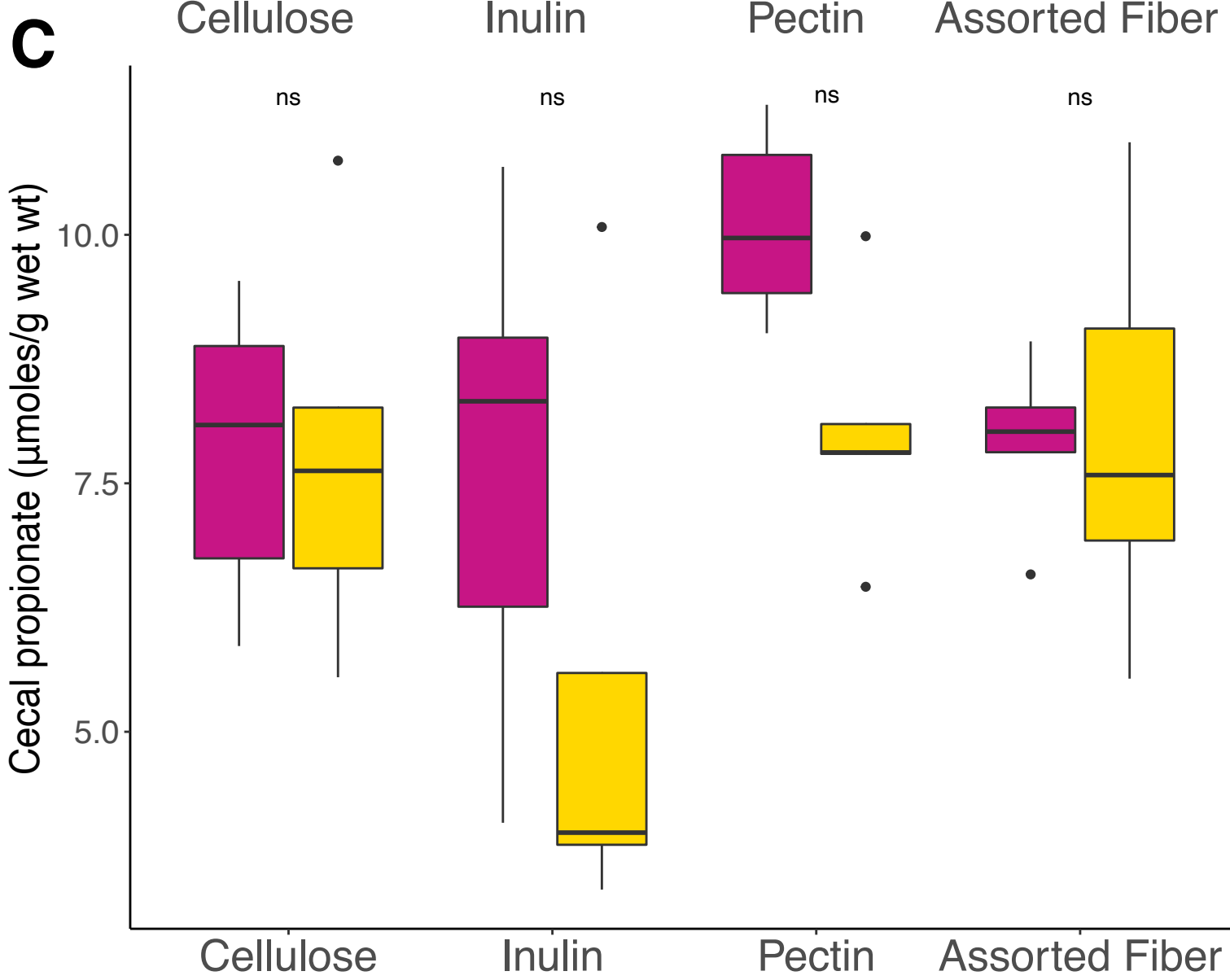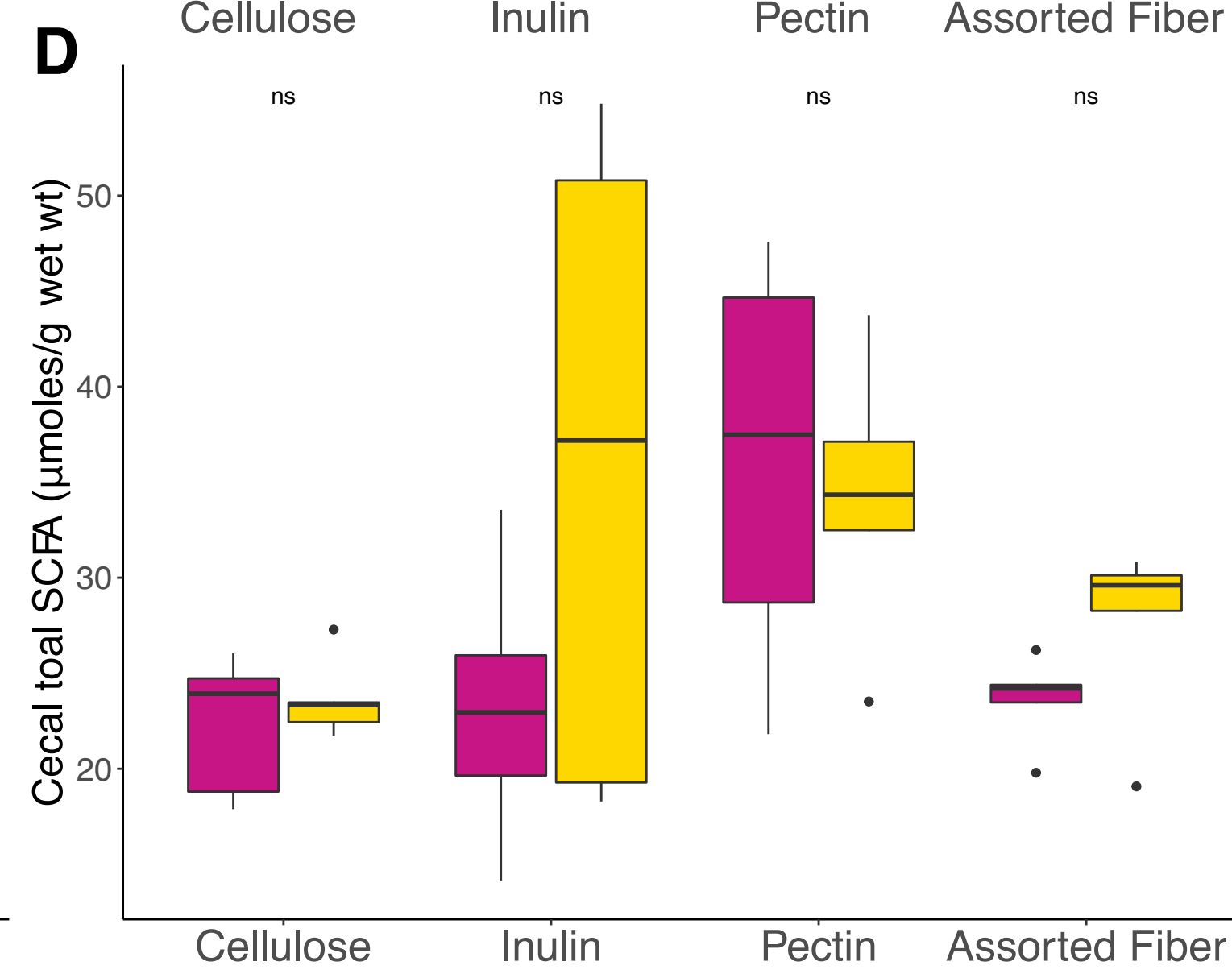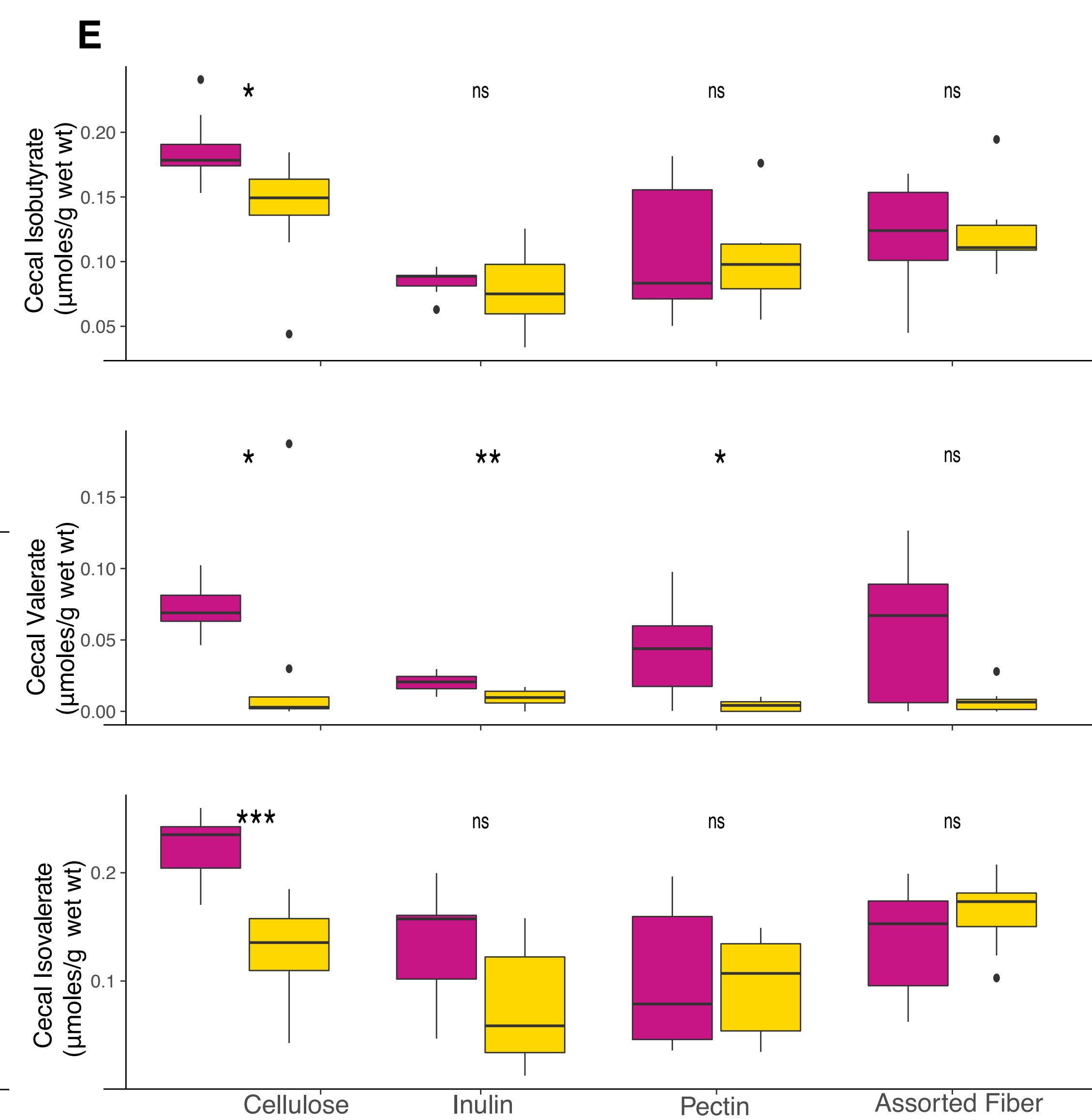

Supplement: Supplementary file 10 — Additional file 9: Fig. S9. Short Chain Fatty Acids (SCFA). Cecal levels of (A) acetate; (B) butyrate, (C) propionate and total SCFA (umoles/g wet wt) E. valerate and Branched-chain Fatty Acids (BCFA) Isobutyrate and Isovalerate of SubA (magenta) and SubB (yellow) by diet. Wilcoxon test comparison *P < 0.05, **P < 0.01, ***P < 0.001, ****P < 0.0001. [file 40168_2021_1061_MOESM10_ESM.pdf]

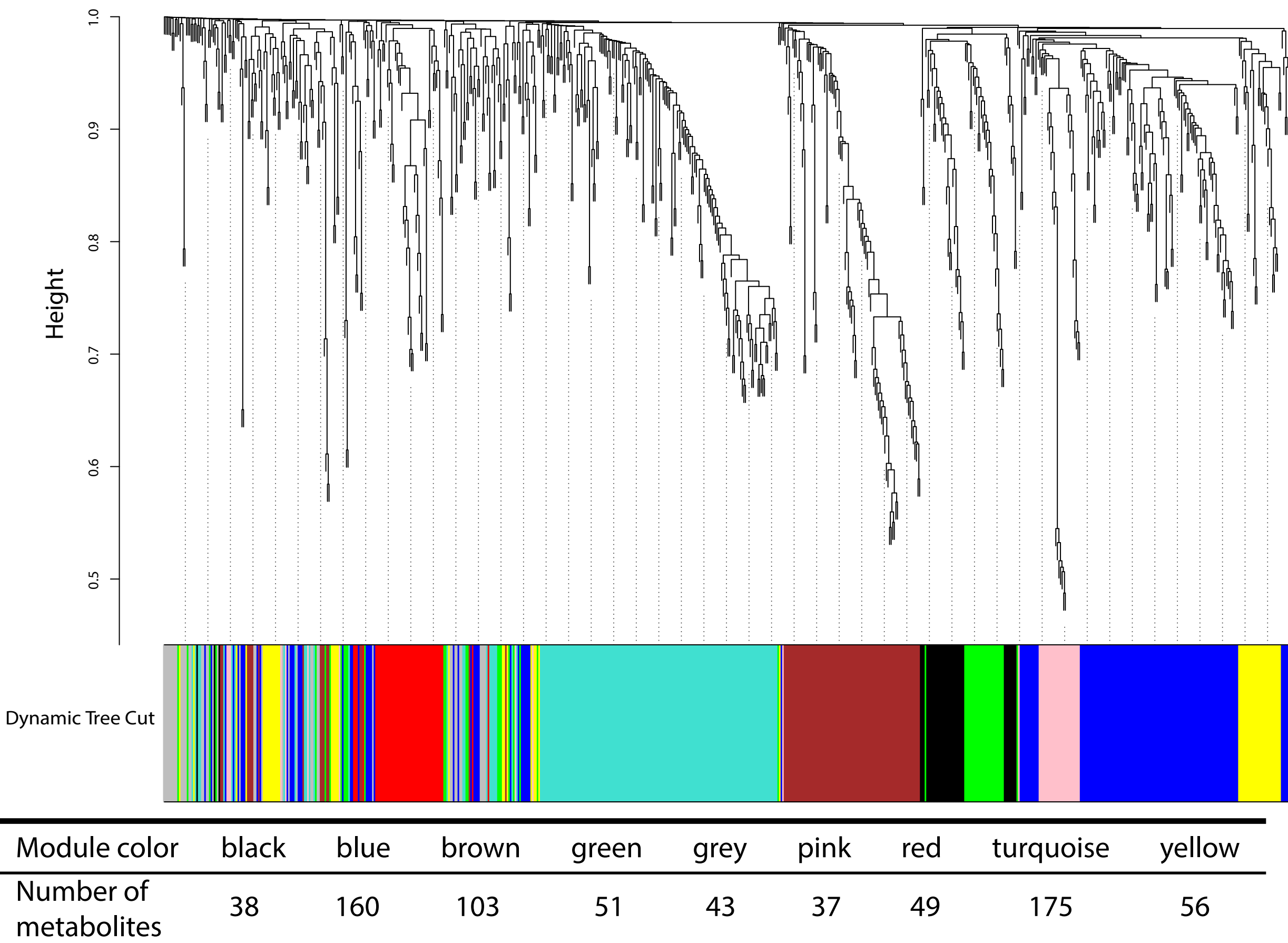

Supplement: Supplementary file 11 — Additional file 10: Fig. S10. Dendrogram of serum metabolites from transplanted mice. Clustering dendrograms of 712 serum metabolites with dissimilarity based on topological overlap, together with assigned module colors. There are 9 modules that cluster different numbers of metabolites. [file 40168_2021_1061_MOESM11_ESM.pdf]

A

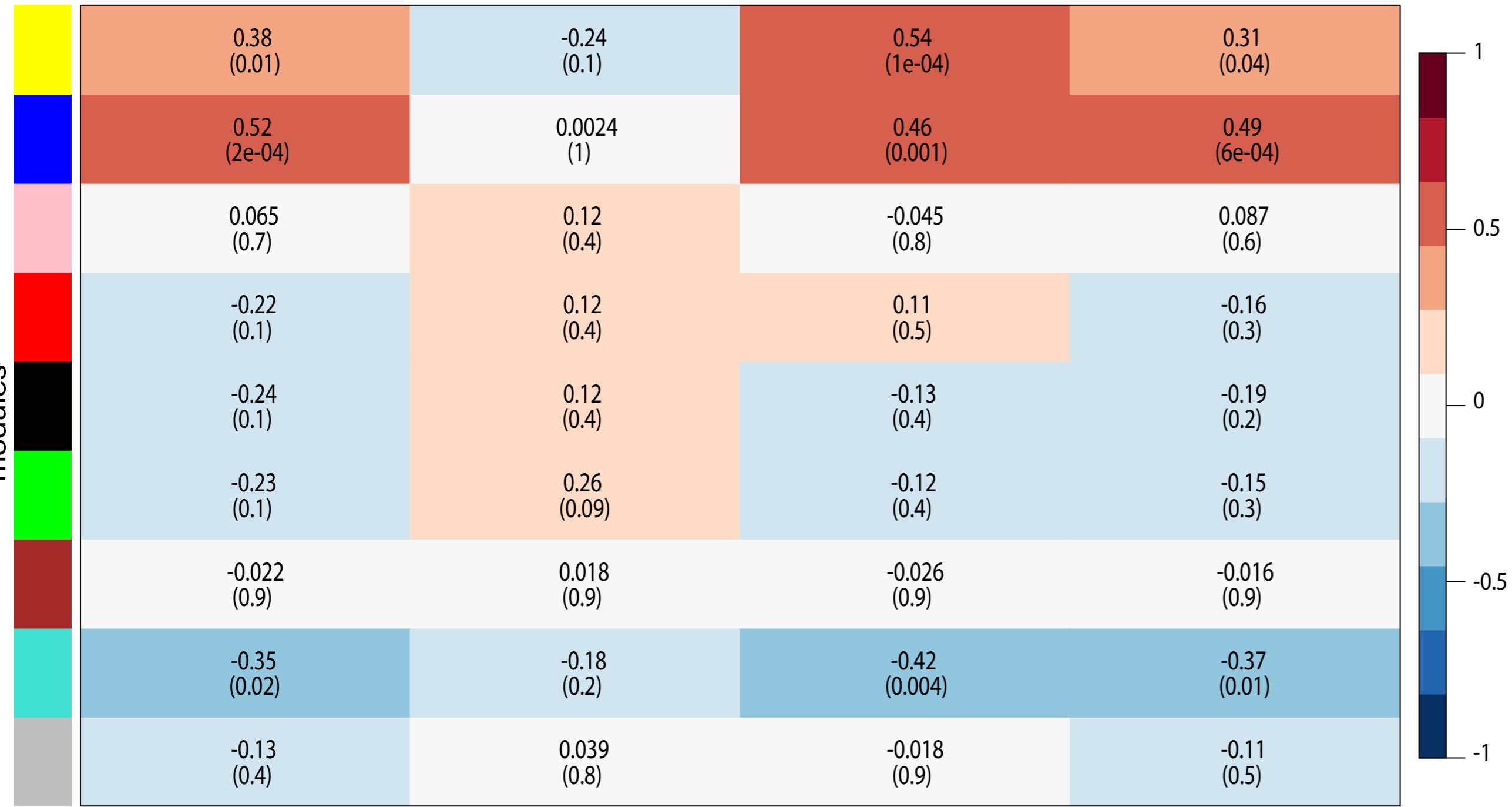

module

Yellow

Blue

Turquoise

B

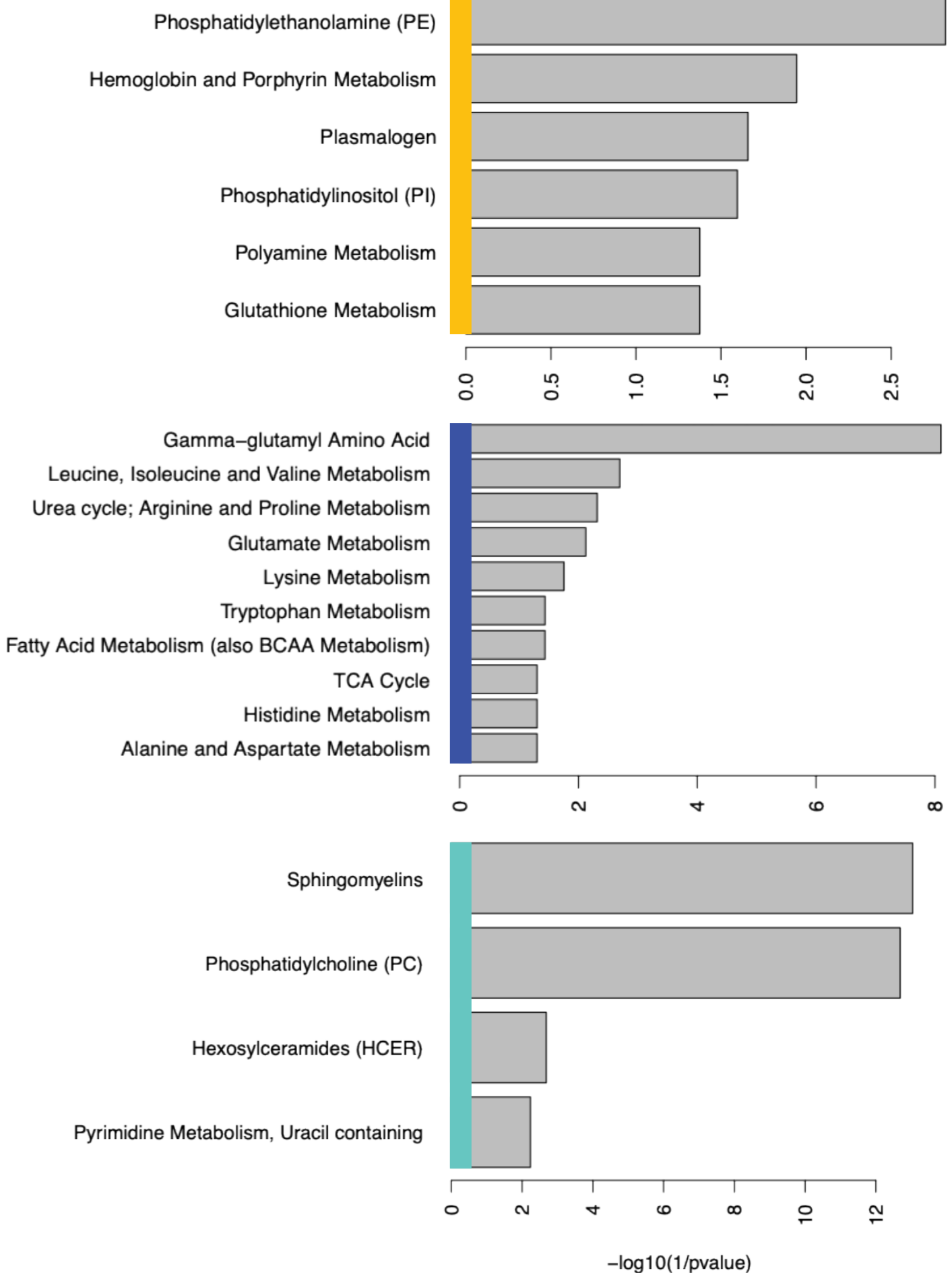

Supplement: Supplementary file 12 — Additional file 11: Fig. S11. Correlations between Short Chain Fatty Acids and blood metabolites. Correlation matrix between metabolites consensus modules and cecal SCFAs from mice described in Fig. 1. Modules were determined based on patterns of co-abundance of metabolites using weighted correlation network analysis (WGCNA). Each of the modules was labelled with a unique color as an identifier. Each module was tested for correlation with each cecal SCFA quantified. Within each cell, upper values are correlation coefficients between module and the phenotypes; lower values are the corresponding FDR adjusted-P values. B. Pathways enriched in the yellow, blue and turquoise modules as determined by Fisher’s test. [file 40168_2021_1061_MOESM12_ESM.pdf]

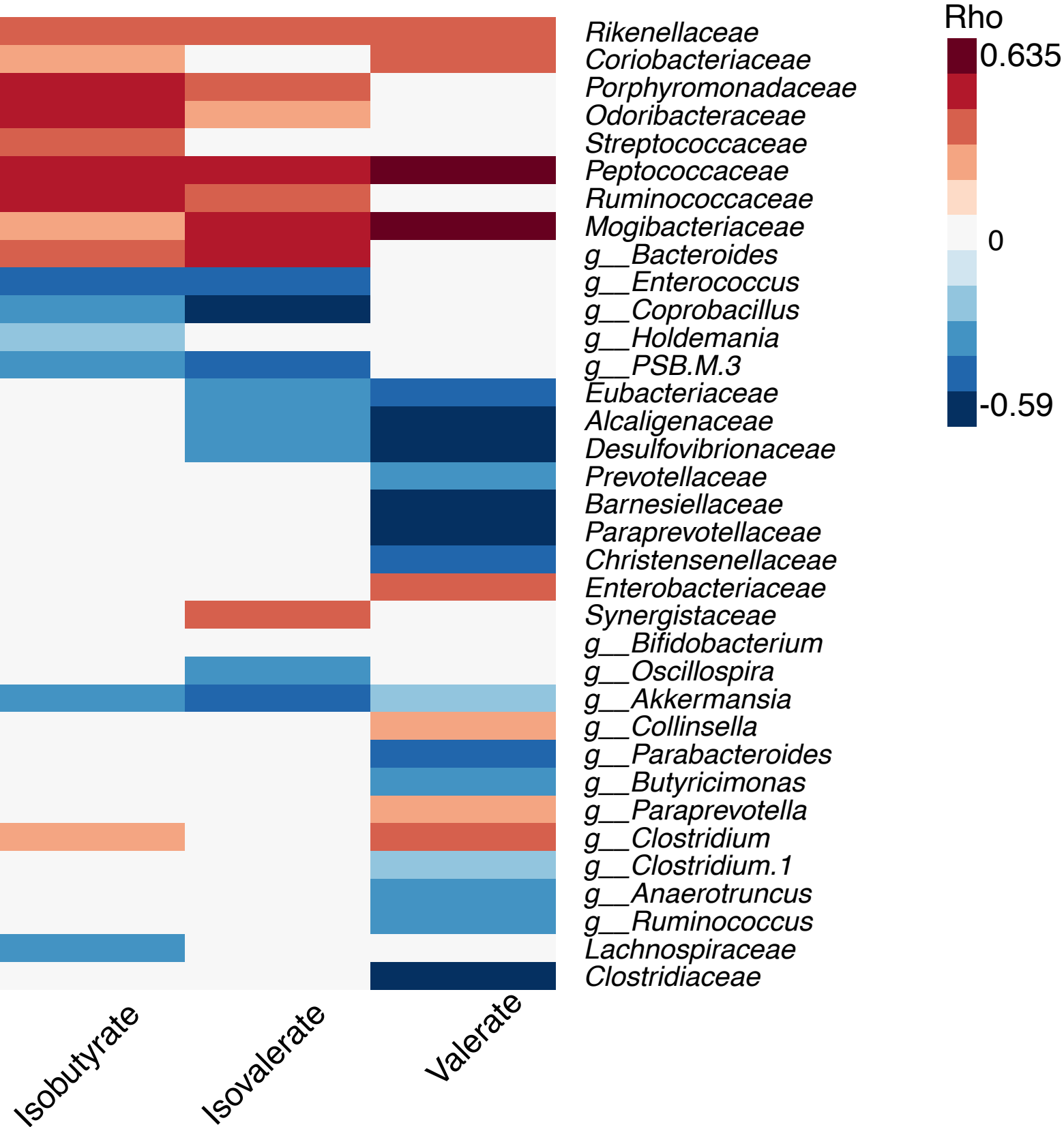

Supplement: Supplementary file 13 — Additional file 12: Fig. S12. Branched-Chain Fatty Acids and taxa correlation. The heatmap shows all correlations (P < 0.05) using Spearman method between BCFA and taxa in each dietary intervention. [file 40168_2021_1061_MOESM13_ESM.pdf]

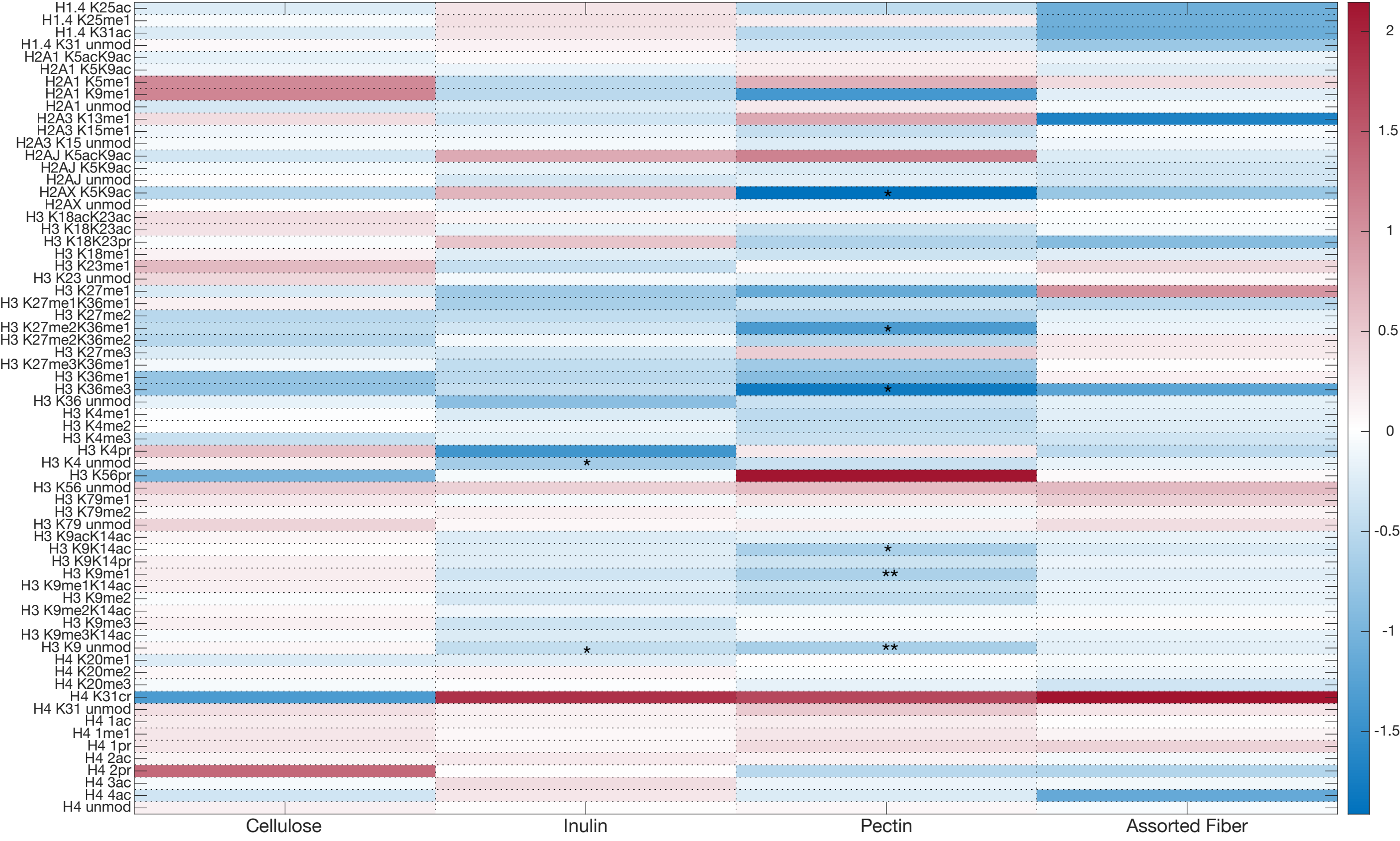

Supplement: Supplementary file 14 — Additional file 13: Fig. S13. Gut community-mediated epigenetic changes in liver are sensitive to dietary fiber. Abundance of histone Post-Translational Modifications (PTMs) on H3 lysines (K9, K14, K27, and K36). A. H3K9K14 peptide. B. H3K27K36 peptide (n = 4/community/diet). *P < 0.05, **P < 0.01; ac, acetylated; unmod, unmodified; meth1,2,3, mono- di- and try- methylated respectively; pr, propionylated. [file 40168_2021_1061_MOESM14_ESM.pdf]

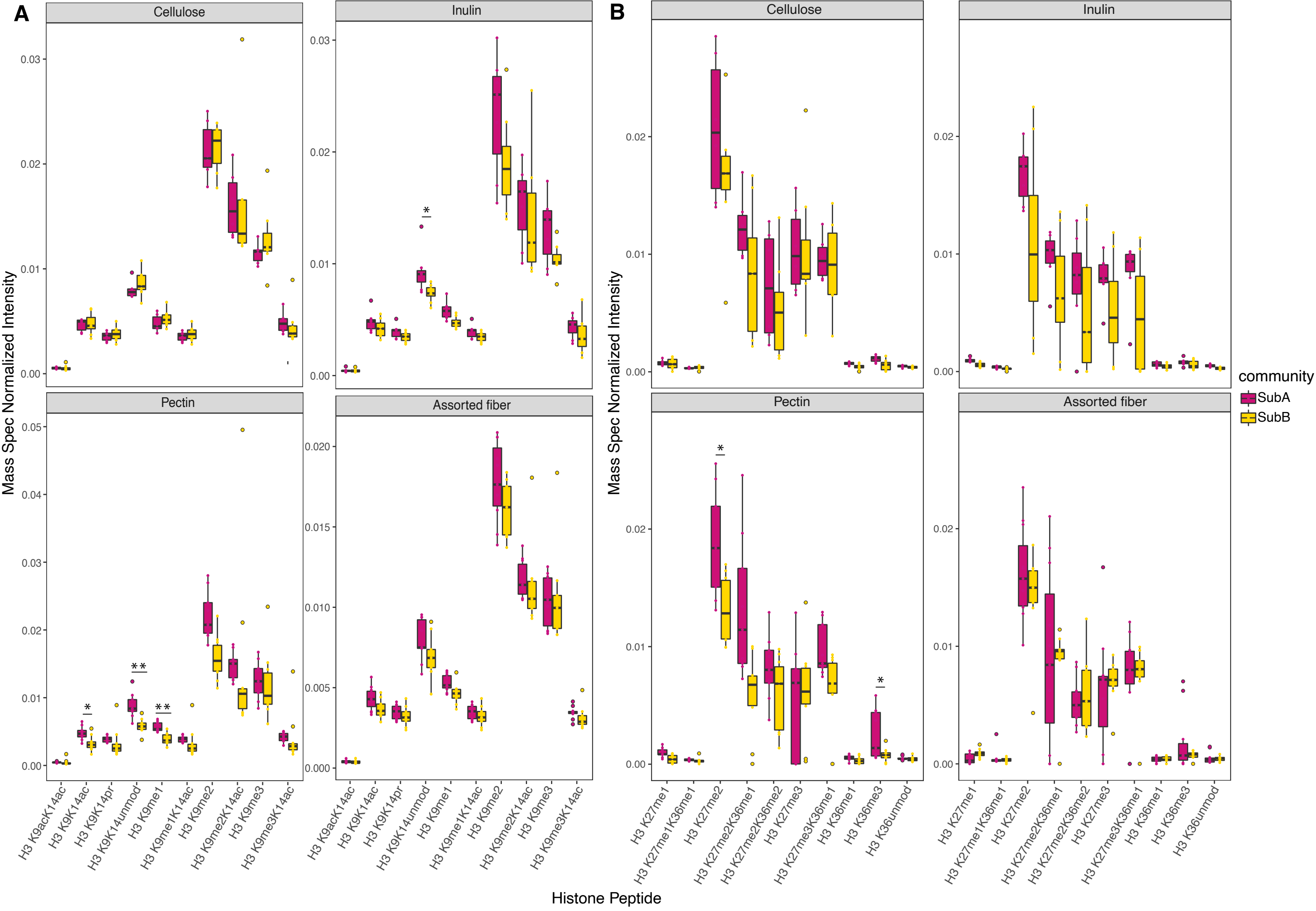

Supplement: Supplementary file 15 — Additional file 14: Fig. S14. Effect of gut community on liver histone post-translational modifications (PTMs). Heatmap show relative difference in abundance for each histone PTM quantified in liver for mice colonized with the two communities (Log2 fold-change of SubB/SubA) in each diet. *P < 0.1, **P < 0.01, ***P < 0.001 (n = 3-4/condition). ac, acetylated; unmod, unmodified; meth1,2,3, mono- di- and try- methylated respectively; pr, propionylated. [file 40168_2021_1061_MOESM15_ESM.pdf]
