# Supplementary material for: Gut microbiome variation modulates the effects of dietary fiber on host metabolism
Source: Microbiome. 2021 May 20;9:117. doi: 10.1186/s40168-021-01061-6 (PMC8138933; doi:10.1186/s40168-021-01061-6)
Supplement: Supplementary file 16 — Additional file 15: Fig. S15. Dendrogram of liver transcripts from transplanted mice. Clustering dendrograms of genes with dissimilarity based on topological overlap, together with assigned module colors. There are 14 modules that cluster different numbers of transcripts. [file 40168_2021_1061_MOESM16_ESM.pdf]

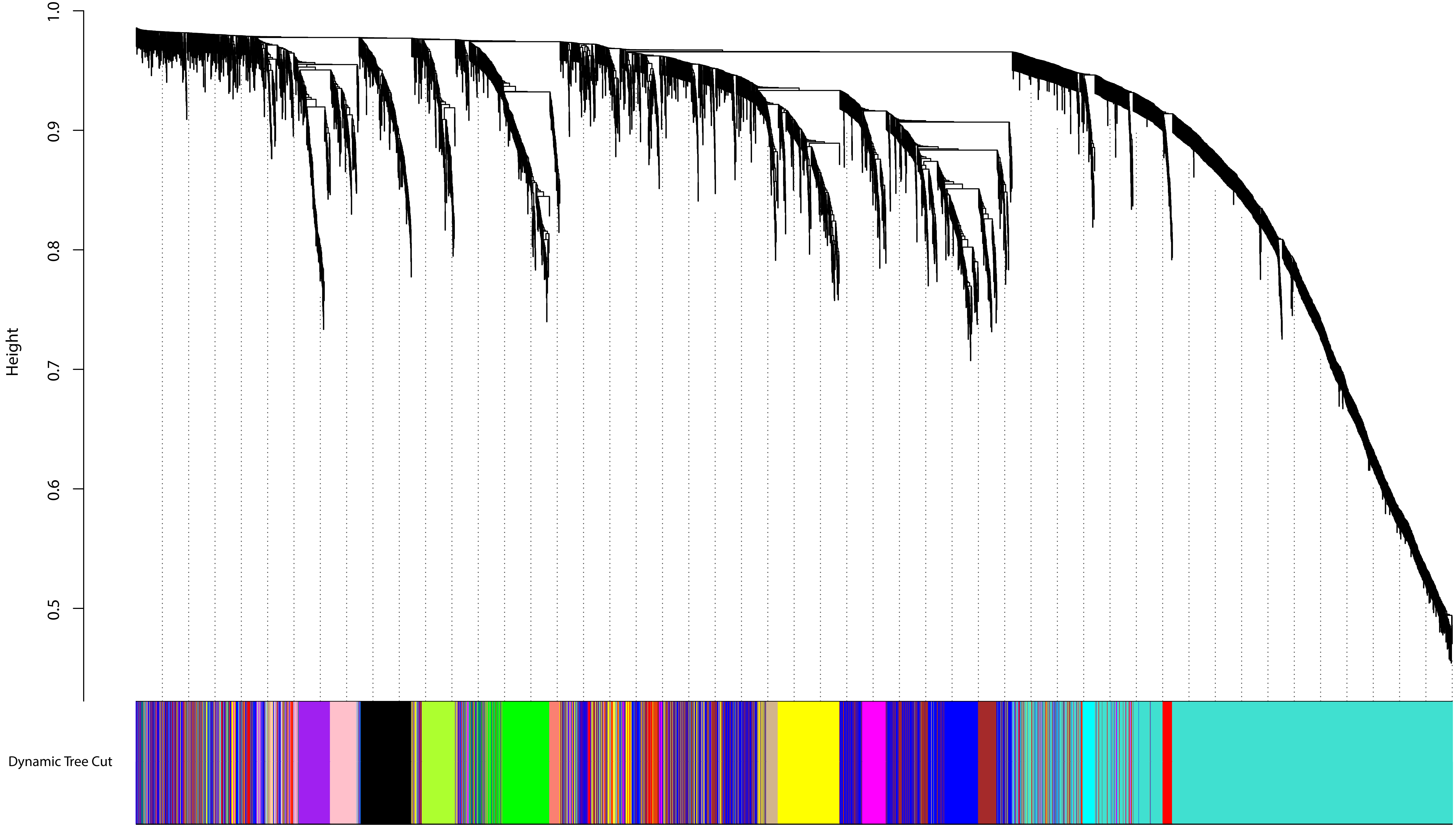

Module color	black	blue	brown	cyan	green	greenyellow	magenta	pink	purple	red	salmon	tan	turquoise	yellow
Number of genes	192	922	542	51	256	124	166	171	152	226	61	109	1572	456
