# Supplementary material for: Gut microbiome variation modulates the effects of dietary fiber on host metabolism
Source: Microbiome. 2021 May 20;9:117. doi: 10.1186/s40168-021-01061-6 (PMC8138933; doi:10.1186/s40168-021-01061-6)
Supplement: Supplementary file 17 — Additional file 16: Fig. S16. Gene Ontology and KEGG pathway enrichment of transcripts in the blue module associated with metabolic phenotypes. A. Biological Process GO and KEGG enrichment of blue module associated with adiposity, B. Association with liver triglycerides. C. Association with glucose. Gene counts and FDR adjusted-P values are indicated for each enrichment box. [file 40168_2021_1061_MOESM17_ESM.pdf]

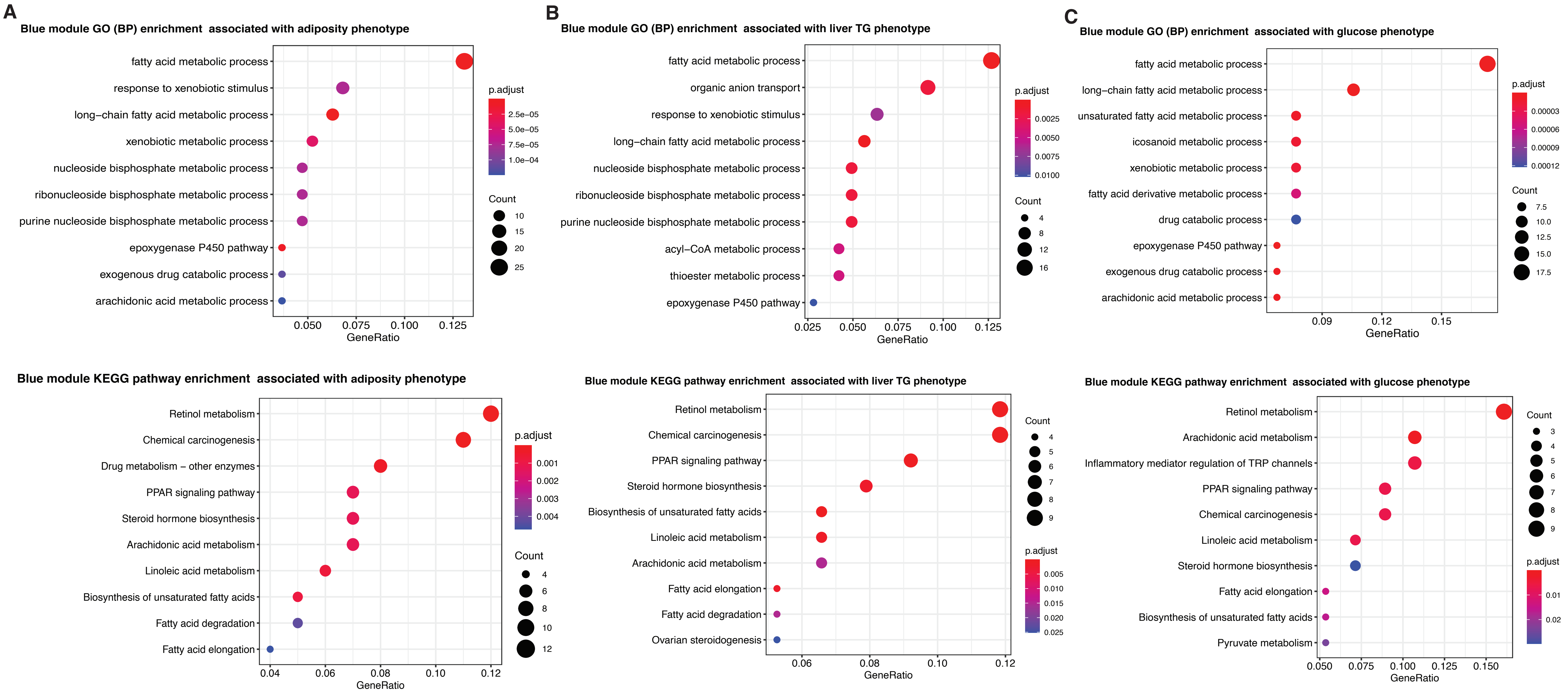
A
Blue module GO (BP) enrichment associated with adiposity phenotype
fatty acid metabolic process
response to xenobiotic stimulus
long−chain fatty acid metabolic process
xenobiotic metabolic process
nucleoside bisphosphate metabolic process
ribonucleoside bisphosphate metabolic process
purine nucleoside bisphosphate metabolic process
epoxygenase P450 pathway
exogenous drug catabolic process
arachidonic acid metabolic process
0.050 0.075 0.100 0.125
GeneRatio
p.adjust
2.5e−05
5.0e−05
7.5e−05
1.0e−04
Count
10
15
20
25
B
Blue module GO (BP) enrichment associated with liver TG phenotype
fatty acid metabolic process
organic anion transport
response to xenobiotic stimulus
long−chain fatty acid metabolic process
nucleoside bisphosphate metabolic process
ribonucleoside bisphosphate metabolic process
purine nucleoside bisphosphate metabolic process
acyl−CoA metabolic process
thioester metabolic process
epoxygenase P450 pathway
0.025 0.050 0.075 0.100 0.125
GeneRatio
p.adjust
0.0025
0.0050
0.0075
0.0100
Count
4
8
12
16
C
Blue module GO (BP) enrichment associated with glucose phenotype
fatty acid metabolic process
long−chain fatty acid metabolic process
unsaturated fatty acid metabolic process
icosanoid metabolic process
xenobiotic metabolic process
fatty acid derivative metabolic process
drug catabolic process
epoxygenase P450 pathway
exogenous drug catabolic process
arachidonic acid metabolic process
0.09 0.12 0.15
GeneRatio
p.adjust
0.00003
0.00006
0.00009
0.00012
Count
7.5
10.0
12.5
15.0
17.5
Blue module KEGG pathway enrichment associated with adiposity phenotype
Retinol metabolism
Chemical carcinogenesis
Drug metabolism – other enzymes
PPAR signaling pathway
Steroid hormone biosynthesis
Arachidonic acid metabolism
Linoleic acid metabolism
Biosynthesis of unsaturated fatty acids
Fatty acid degradation
Fatty acid elongation
0.04 0.06 0.08 0.10 0.12
GeneRatio
p.adjust
0.001
0.002
0.003
0.004
Count
4
6
8
10
12
Blue module KEGG pathway enrichment associated with liver TG phenotype
Retinol metabolism
Chemical carcinogenesis
PPAR signaling pathway
Steroid hormone biosynthesis
Biosynthesis of unsaturated fatty acids
Linoleic acid metabolism
Arachidonic acid metabolism
Fatty acid elongation
Fatty acid degradation
Ovarian steroidogenesis
0.06 0.08 0.10 0.12
GeneRatio
Count
4
5
6
7
8
9
p.adjust
0.005
0.010
0.015
0.020
0.025
Blue module KEGG pathway enrichment associated with glucose phenotype
Retinol metabolism
Arachidonic acid metabolism
Inflammatory mediator regulation of TRP channels
PPAR signaling pathway
Chemical carcinogenesis
Linoleic acid metabolism
Steroid hormone biosynthesis
Fatty acid elongation
Biosynthesis of unsaturated fatty acids
Pyruvate metabolism
0.050 0.075 0.100 0.125 0.150
GeneRatio
Count
3
4
5
6
7
8
9
p.adjust
0.01
0.02
