# Supplementary material for: Gut microbiome variation modulates the effects of dietary fiber on host metabolism
Source: Microbiome. 2021 May 20;9:117. doi: 10.1186/s40168-021-01061-6 (PMC8138933; doi:10.1186/s40168-021-01061-6)
Supplement: Supplementary file 30 — Additional file 29. Supplemental results. Effects of microbiota-fiber interactions on liver histone posttranslational modifications. [file 40168_2021_1061_MOESM30_ESM.docx]

**Supplemental results**

*Effects of Microbiota-fiber interactions on liver histone posttranslational modifications.* Epigenetic states of chromatin are reflected in the covalent posttranslational modifications (PTMs) on histone proteins. The N-terminal tail of histones is highly decorated with PTMs, including lysine acetylation and methylation. These PTMs are combinatorial and are thought to integrate multiple environmental signals, including microbial metabolites [1–3]. We examined histone PTM states as a function of community for each fiber treatment. We surveyed >60 acetylated, propionylated, and methylated histone PTM states in liver samples [4]. The liver receives a large fraction of its blood supply through the portal circulation, which is the direct venous outflow of the intestine. As such, the liver is continuously exposed to gut microbial-derived products, including SCFAs and bacterial toxins [5]. We found that colonization of mice with SubA and SubB communities affected liver histone acetylation and methylation to different extents in the four fiber groups. The most significant changes were observed when comparing the effects of pectin between the two communities (Fig. S13). Within the family of H3K9K14 peptides quantified there is significant loss of unmodified, monoacetylation, and monomethylation peptides (Fig. 14A) that is likely accounted for by an increase in H3K9me3, though the errors on this peptide species are too large to confirm it. The H3K27K36 family of peptides shows a net loss of K36 methylation but a trending gain of tri-methylation at H3K27 (Fig. S13 and Fig. 14B). Collectively, these specific changes suggest a net increase in repressive chromatin states in SubB- relative to SubA-colonized mice in the pectin diet. Perhaps most surprising is the lack of significant increases in many acetylation sites (Fig. S13); since butyrate is known to be a histone deacetylase inhibitor, we had hypothesized that butyrate levels would primarily influence histone acetylation. However, these results suggest that gut microbiota-fiber interactions impact histone modifications in a complex fashion that cannot be solely isolated to the capacity to generate butyrate.

**References**

1. Fan J, Krautkramer KA, Feldman JL, Denu JM. Metabolic Regulation of Histone Post-Translational Modifications. ACS Chem Biol. 2015;10:95–108.

2. Krautkramer KA, Rey FE, Denu JM. Chemical signaling between gut microbiota and host chromatin: What is your gut really saying? Journal of Biological Chemistry. 2017;292:8582–93.

3. Krautkramer KA, Kreznar JH, Romano KA, Vivas EI, Barrett-Wilt GA, Rabaglia ME, et al. Diet-Microbiota Interactions Mediate Global Epigenetic Programming in Multiple Host Tissues. Molecular Cell. 2016;64:982–92.

4. Krautkramer KA, Reiter L, Denu JM, Dowell JA. Quantification of SAHA-Dependent Changes in Histone Modifications Using Data-Independent Acquisition Mass Spectrometry. J Proteome Res. 2015;14:3252–62.

5. Son G, Kremer M, Hines IN. Contribution of gut bacteria to liver pathobiology. Gastroenterol Res Pract. 2010;2010.
